# Anti-glioma effect of ginseng-derived exosomes-like nanoparticles by active blood–brain-barrier penetration and tumor microenvironment modulation

**DOI:** 10.1186/s12951-023-02006-x

**Published:** 2023-08-04

**Authors:** Jisu Kim, Ying Zhu, Sunhui Chen, Dongdong Wang, Shuya Zhang, Jiaxuan Xia, Shiyi Li, Qiujun QIU, Hyukjin Lee, Jianxin Wang

**Affiliations:** 1 Department of Pharmaceutics, School of Pharmacy, Fudan University and Key Laboratory of Smart Drug Delivery, Ministry of Education, Shanghai, 201203 People’s Republic of China; 2 Department of Oncology, Shanghai Medical College, Fudan University, Shanghai, 200032 People’s Republic of China; 3 Department of Pharmacy, Fujian Provincial Hospital, Fuzhou, 350001 People’s Republic of China; 4 Department of Radiology, Huashan Hospital, Fudan University, Shanghai, 200040 People’s Republic of China; 5 College of Pharmacy, Graduate School of Pharmaceutical Sciences, Ewha Womans University, Seoul, 03760 Republic of Korea; 6 Institutes of Integrative Medicine, Fudan University, Shanghai, 201203 People’s Republic of China

**Keywords:** Ginseng-derived exosome-like nanoparticles, Plant-derived exosome-like nanoparticles, Anti-glioma, Tumor microenvironment, Ginseng

## Abstract

Inhibition of tumor growth and normalization of immune responses in the tumor microenvironment (TME) are critical issues for improving cancer therapy. However, in the treatment of glioma, effective nanomedicine has limited access to the brain because of the blood–brain barrier (BBB). Previously, we demonstrated nano-sized ginseng-derived exosome-like nanoparticles (GENs) consisting of phospholipids including various bioactive components, and evaluated anti-tumor immune responses in T cells and Tregs to inhibit tumor progression. It was found that the enhanced targeting ability of GENs to the BBB and glioma induced a significant therapeutic effect and exhibited strong efficacy in recruiting M1 macrophage expression in the TME. GENs were demonstrated to be successful candidates in glioma therapeutics both in vitro and in vivo, suggesting excellent potential for inhibiting glioma progression and regulating tumor-associated macrophages (TAMs).

**Supplementary Information:**

The online version contains supplementary material available at 10.1186/s12951-023-02006-x.

## Introduction

Glioma is one of the most fatal diseases in the world. According to 2020 statistics on brain tumors in the United States supported by the American Society of Clinical Oncology, the 5-year survival rate for people with cancerous brain tumors was only 6.8% [1]. Due to the location of the tumor growth, the treatment of glioma is very challenging. Furthermore, the brain is protected by the blood brain barrier (BBB) at the boundary between the circulating blood and the extracellular space, which helps guard the brain from common bacterial infections. Therefore, the successful delivery of chemotherapeutic drugs into the brain has been a challenge.

To address this problem, studies have been conducted to develop drug delivery systems for brain tumors. Artificial nanoparticles, such as polymer-based nanoparticles, gold/silver nanoparticles, liposomes, and micelles, have been studied widely due to their cost-effectiveness, ease of modification, and improved brain distribution [2–7]. However, neurotoxicity, inflammation, and immunogenicity are still observed as drawbacks [8–11].

In recent years, the use of biomimetic natural nanoparticles such as mammalian cell-derived exosomes (MDEs) and cell-derived membranes to treat various diseases has attracted attention due to their specific tissue targeting, high biocompatibility, and limited cytotoxicity [12, 13]. The components and biological activity of MDEs depend on the donor cells through which MDEs may serve different functions and therapeutic purposes. According to a previous report by Qian et al., glioma-derived exosomes that deliver bioactive molecules to the BBB and glioma showed very encouraging therapeutic effects [14]. Glioma-derived exosomes with miR-1246-mediated targeting induced M2 polarization and targeted TERF2IP, upregulated the STAT3 signaling pathway, and inhibited the NF-κB signaling pathway. Even though the potential benefits of glioma cell-derived exosomes were exhibited, fundamentally, MDEs have serious challenges including: (i) low production yield, (ii) time-consuming and laborious production process, (iii) difficulty in yielding massive and uniform exosomes, and (iv) immunogenicity and toxicity of MDEs or the various cargo depending on the cellular sources [15–17]. To overcome these drawbacks, there is an urgent need to establish high-yielding and biocompatible nanoparticles [18, 19].

Recently, plant-derived exosome-like nanoparticles (PENs) from a carrot, apple, broccoli, ginger, grape, grapefruit, and cabbage have been developed and evaluated for their characteristics, components, functions, and therapeutic effectiveness [20–26]. Various effects of PENs were reported, including the prevention of liver damage through the interaction with mammalian cells [22], the amelioration of the promotion of DSS-induced intestinal colitis through oral administration, and the reduction of cancer growth through the regulation of macrophage and tumor-associated microenvironments (TAMs) [27–29].

Even though previous studies on PENs indicated anti-inflammatory properties in inflammatory bowel disease and anti-tumor effects in lung cancer, colorectal cancer, and leukemia, the action mechanisms of PENs, including their specific roles and regulation through downstream signaling pathways in disease conditions, have not been clearly elucidated yet, which hinders their application.

*Panax ginseng* C.A. Meyer has been used as a traditional herbal medicine for thousands of years and has been reported to exhibit various pharmacological properties for cardiovascular diseases, neuronal diseases, and cancer [30, 31]. Furthermore, it has been discovered that it has great potential for relieving stress, hypertension, and neurological disorders such as Parkinson's disease and Alzheimer's disease, as well as having an anticancer effect on glioma [32–34].

Exosome-like nanoparticles can also be isolated from *Panax ginseng*. Given the potent pharmacological activities of the components of *Panax ginseng*, it can be anticipated that the ginseng-derived exosome-like nanoparticles (GENs) will contain various bioactive compounds such as ginsenosides, miRNAs, proteins, and lipids [35], thus holding potential in the treatment of various diseases, such as cancers.

According to several previous reports [36–38], GENs have been found to be beneficial in immune regulation as a potential treatment for cancer and damaged tissue. Han et al. [36] reported that biologically functional GENs enhance the efficacy of immune checkpoint antibodies by switching cold TAMs to hot TAMs. GENs could be used as a part of a combinational adjuvant strategy with PD-1 monoclonal antibodies in colon cancer models *in viv*o. Xu et al. [38] reported that GENs serve as nanoplatforms for deliverying nucleic acids to mammalian cells. They show great potential in improving cutaneous wound healing through the targeting genes such as Tmem100 and Vrk1 in bone marrow-derived mesenchymal stem cells (BMSCs). GENs also stimulate neural differentiation, maturation, and sensory function recovery of BMSCs.

However, the applications of GENs remain limited due to a lack of understanding of the mechanisms of actions of GENs and their components in diseases, such as in cancer and tumor microenvironments (TMEs), requiring further in-depth studies.

When treating a brain tumor, BBB penetration is crucial [39, 40]. The BBB maintains brain homeostasis of the central nervous system (CNS) under normal conditions and protects the sensitive environment of the brain by impeding the transport of toxic drugs and inflammatory infections. However, the BBB is formed by endothelial cells that are closely connected by tight and adherens junction molecules, which significantly reduces the effectiveness of drugs targeting brain tumors. Therefore, there has been increased attention on the development of drugs with the potential to target and cross the BBB.

In this study, we developed GENs using a simple and eco-friendly method to identify their components, including genes, lipids, proteins, and metabolites, and evaluated their therapeutic effects on brain tumors in vivo. We observed an improved targeting effect of nano-sized GENs, carrying various chemical cargoes, on C6 glioma cells by crossing the BBB. Furthermore, we analyzed the composition of GENs and found that GENs mainly consisted of phosphatidylcholines (PC), along with 98 miRNAs and 86 proteins similar to those of *Panax ginseng*. GENs exhibited high stability, and upon injection, they were taken up by the brain tumor by passing through the BBB. Furthermore, we found that ptc-miR396f-mediated targeting of apoptosis-related genes in GENs exerted a significant gene silencing effect on the c-MYC, resulting in the suppression of tumor growth and prolonged survival rate in a glioma-bearing mice in vivo. We observed the downregulation of pro-tumoral cytokines, induction of T cells, suppression of regulatory T cells (Tregs) in TMEs, and orchestrated activation of CAFs by TMEs.

Collectively, the findings of the study elucidated the glioma inhibition mechanisms by which GENs inhibit glioma and highlighted their therapeutic potential in regulating TMEs.

## Materials and methods

### Chemicals and reagents

1,1’-dioctadecyl-3,3,3’,3’–tetramethylind-odicarbocyanine, 4-chlorobenzenesulfonate salt (DiD) and 1,1’-dioctadecyl-3,3,3’,3’-tetramethylindotricarbocyanine iodide (DiR) were purchased from Fanbo Biochemical Co., Ltd. (Beijing, China). D-luciferase ((S)-2-(6-Hydroxy-2-benzothiazolyl)-2-thiazoline-4-carboxylic acid potassium salt, 4,5-Dihydro-2-(6-hydroxy-2-benzothiazolyl)-4-thiazolecarboxylic acid potassium salt, Firefly luciferin potassium salt) was purchased from Sigma-Aldrich. (St. Louis, USA) Cholesterol was purchased from Sinopharm Chemical Reagent Co., Ltd. (Shanghai, China). Egg yolk phospholipid (EPC) was provided by A.V.T Pharmaceutical Co., Ltd. (Shanghai, China).

### Isolation and separation of the ginseng derived exosome

10–15 g of four-year-old-grade ginsengs harvested from Northeastern China were purchased from a local market. (Jilin, China) The ginsengs were washed with water to remove dirt and contaminants. After washing, the ginsengs were thoroughly ground in 30 mL of 1X PBS with a high-speed blender for 10 min (pause 1 min after 1 min blending). The ginseng juice was filtered and squeezed on a sieve to remove rough debris. The collected juice was centrifuged at 2000 g for 20 min and the supernatant was then transferred and centrifuged at 10,000 g for 60 min. The supernatant was filtered at 0.2 μm of a pore filter (Acrodisc® Syringe Filters with Supor® Membrane, PALL, USA). The filtered juice was transferred to the top of sucrose cushion layers (27, 68% of sucrose) and then centrifuged at 100,000*g* for 90 min (Hitachi Ultracentrifuge CP100NX, Himac, Japan). The cushion layer was obtained and purified using sucrose gradients (8, 30, 45, and 60%, respectively) at 200,000*g* for 90 min to isolate the exosome by its density. GENs at between 8 and 30% interface were harvested. All procedures of the ultracentrifugation were set at 4 ℃. The concentration of the exosome was expressed as protein concentration by the BCA protein assay kit (Beyotime, Shanghai, China) and determined by Nanoparticle tracking analysis (Malvern Instrument, UK).

### Size distribution and zeta potential analysis

The nanoparticle size and surface charge were evaluated using a Zetasizer Nano ZS (Malvern Instrument, UK) as follows. The ginseng derived exosome was diluted to a final dilution of 1:10 and 1 mL of the diluted sample was analyzed three times at 37 ℃.

### Measurement of total RNA and protein concentration of GENs

Total RNA was extracted from GENs using TRIzol reagent according to the manufacturer’s instruction. The total RNA was quantified using a UV–Vis spectrophotometer at 260 nm. (NanoDrop 2000, thermo scientific, USA) The protein concentration of freeze-dried GENs dissolved with 1X phosphate-buffered saline (PBS) was measured using Bradford assay kit according to the manufacturer’s instruction.

### Cell viability by MTT assay

Approximately 2 × 10^5^ cells/mL were seeded into 96-well culture plates and treated with or without GENs at 48 h. After treatment, 50 μL of the new media was replaced and continuously 50 μL of MTT solution (2 mg/mL) was added into the wells and the well plate was incubated at 37 °C for 4 h while avoiding light. The solution was removed completely and 150 μL of DMSO was added to solubilize the formazan product and incubated for 15 min at 200 rpm at room temperature. The absorbency at 570 nm was measured using a Bio-Rad micro-plate reader. Cell viability was calculated according to the following equation.

Cell viability (%) = $$\frac{{\mathrm{OD}}_{\mathrm{sample}}-{\mathrm{OD}}_{\mathrm{blank}}}{{\mathrm{OD}}_{\mathrm{control}}-{\mathrm{OD}}_{\mathrm{blank}}}$$  × 100%

### Cellular apoptosis assay by flow cytometry

Cellular apoptosis assay was tested with the Annexin V-FITC kit according to the manufacturer’s instructions. After 24 h of incubation of GENs ranging from 0.97 to 62.5 μg/mL, C6 glioma cells were washed twice with cold PBS, digested, and harvested. After centrifugation three times for washing, then the cells were dissolved into the binding buffer. Annexin V-FITC and PI were added (BioVision, Milpitas, CA, USA), and the cells were incubated for 10 min at room temperature in the dark. 300 μL of the stained cells in binding buffer was transferred to a 96-well plate and the cells were analyzed by flow cytometry. (FACScan, BD, Germany).

### Real time PCR assay

Total RNA was harvested from C6 cells treated with a serial dilution of GENs ranging from 0 µg/mL to 50 µg/mL for 24 h and 48 h. The total RNA was conducted to synthesize complementary DNA and consistently RT-PCR was performed using Hifair III One Step RT-qPCR SYBR Green Kit (Yeasen, China) according to the manufacturer’s instructions. To determine apoptosis of the tumor cell, apoptosis-related genes were examined including BAX, BCL-2, BCL-XL, Survivin and the reference gene, GAPDH. To evaluate the gene expression of the targets, the solution containing DNA polymerase, primer pair and cDNA was pre-heated for 5 min at 95˚C to activate and denature non-specific DNA binding. Immediately, the reaction was performed by 40 cycles of 95˚C for 20 s, 55˚C for 30 s and 68˚C for 20 s using QuantStudio 3. Relative expression related to cell apoptosis was calculated using the ΔΔCT method and normalized to the expression of GAPDH as a standard for gene expression quantification. All qRT-PCRs were performed in triplicate, and the data are presented as means ± standard errors of the means (S.E.M).

When using the fresh brains, after five times of injections of GENs, the mice were sacrificed and perfused with 30 mL of 1X PBS and then the fresh brains were extracted on the same day (N = 3, each group). The brain was finely minced with 5 mL of DMEM and centrifuged at 600 g for 5 min. After centrifugation, the clear supernatant was transferred to a new tube. Total RNA was extracted total RNA using a total RNA extraction kit (Yeasen, China) as the product manual. Finally, the total RNA was synthesized into complementary DNA and RT-PCR was performed as same as the protocol mentioned above. CD86 and IL6 were used as M1 macrophage markers and CD206 and IL10 were used as M2 macrophage markers. Plus, F4/80 was used as a pan macrophage marker. GAPDH served as a Housekeeping gene.

### Animal experiment

Male Wistar rats (8 weeks old, 200–250 g) and male Balb/C mice (6–8 weeks old, 18-20 g) were purchased from Sino-British SIPPR/BK Lab. Animal Co., Ltd. (Shanghai, China). The rats were under standard conditions of 20 ± 1℃, a 12:12 h light and dark cycle, and fed an unrestricted regular diet and water. All animal experiments were performed at Fudan university following the Guiding Principles for the Care and Use of Experimental Animals (Shanghai, China). Wistar rats and Balb/C mice were used to establish the brain tumor model. Wistar rats were orthotopically injected with 5 µL of 1 × 10^8^ cells/mL C6 glioma into the brain of the Wistar rats. On day 14 post-tumor implantation, tumor-bearing rats were randomly separated into two groups; the control group (N = 6) and the IV injcetion group (N = 6) (N = 6). The Wistar rats were treated with 1 mL of 2 mg of GENs (based on the protein concentration) and PBS by IV administration every other day for 7 times. After 7 times of IV injections of GENs, the rats in the control group and IV injection group were imaged by MRI imaging.

Balb/C mice were prepared in the same way. Balb/C mice were treated with 5 µL of 2 mg of GENs (based on the protein concentration) by IC administration and 200 µL of 2 mg of GENs (based on the protein concentration) and PBS by IV administration every other day 7 times.

A median survival rate of mice was monitored for 110 days in C6 glioma cell bearing mice. A Kaplan–Meier method was used to estimate the survival probability, and the log-rank test was used to compare the fraction of surviving mice between treatment groups.

### Preparation of DiD-labeled GENs and in vivo imaging of targeting effect and organ distribution ex vivo of GENs

To determine in vivo organ distribution of GENs, DiD-labeled GENs were prepared. 4 μL of 1 mg/mL of DiD-lipophilic dye, 250 μL of 1 mg/mL of GENs and 50 μL of 1X PBS were mixed and incubated at room temperature for 30 min. Then, the solution was centrifuged using UFC5100 Amicon Ultra-0.5 Centrifugal Filter Unit, 100 kDa (USA, Merck) to remove free DiD dye. The fluorescence intensity of DiD-labeled GENs was measured at 5 × 10^8^ using In Vivo Imaging System Spectrum (IVIS) and 200 μL of DiD-labeled GENs were injected into Balb/C mice. The targeting effect of GENs in the mice was imaged at different time points. After 24 h injection of DiD-labeled GENs, the mice was sacrificed and the organs were dissected. The organs were treated with 4% paraformaldehyde and visualized using the IVIS Spectrum system.

### Statistical analysis

All statistical analysis in this study was presented as values with a standard deviation. (As the mean ± SD) The statistical mean differences were achieved with unpaired t-test or one-way ANOVA to assess the significance and all statistical methods were performed using GraphPad Prism 7.0 software (CA, USA). The unpaired t-test was used for statistical analyses applied to two groups separately. The percentage of apoptotic cells in GENs was determined in a concentration-dependent manner compared to the negative control and was also statistically analyzed by the unpaired t-test. In vivo assay, statistical analysis of the comparison of gene expressions and α-SMA between GENs’ treatment groups and the control group was performed by one-way ANOVA. A P < 0.05 was considered statistically significant, and non-significant result were recorded as N.S.

## Results

### Isolation, characterization, and stability of GENs

**Fig. 1:**
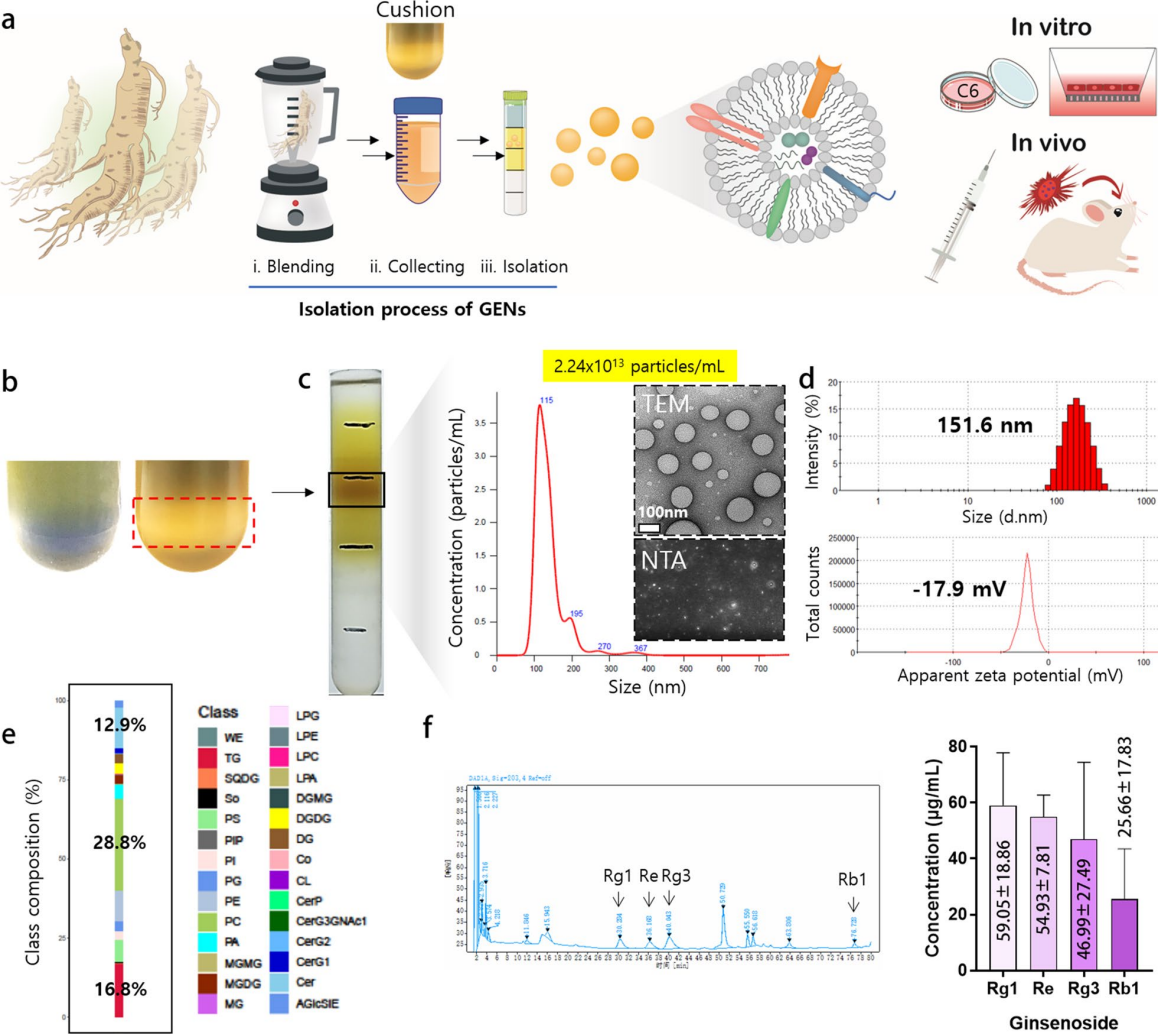
Isolation and characterization of ginseng-derived exosome-like nanoparticles (GENs). **a** A schematic illustration of the isolation process of GENs from fresh ginseng. Ginseng juice was sequentially centrifuged at low and high velocity of speed to isolate GENs. The sucrose cushion method was employed by layering sucrose at concentrations of 68% and 27%. GENs were purified by a buoyant density from 1.13 to 1.19 g/mL using a density gradient method. After the purification of GENs, the cellular toxicity and therapeutic efficiency of GENs were evaluated by in vitro and in vivo analyses. **b** The sucrose cushion method to prevent disruption of the extracellular vesicles (EVs) and contaminants caused by excessive aggregates of EVs during centrifugation (right). **c** The specific layer between 8 and 30% of sucrose was achieved and the concentration of 2.24 × 10.^13^ particles/mL and size distribution were determined with a serial dilution of GENs by nanoparticle tracking analysis (NTA). The round-shaped morphology of GENs was observed by transmission electron microscopy (TEM; inset). **d** The size and zeta potential of GENs were measured at 37 ℃ by dynamic light scattering (DLS). **e** A graph of lipidomic analysis was reported as the percentage of the components of lipids of GENs. **f** The concentration of ginsenosides of GENs was determined by high performance liquid chromatography (HPLC). wax esters (WE), triglycerides (TG), sulfoquinovosyldiacylglycerol (SQDG), sphingosine (So), phosphatidylserine (PS), phosphatidylinositol phosphate (PIP), phosphatidylinositol (PI), phosphatidylgylcerols (PG), phosphatidylethanolamine (PE), phosphatidylcholine (PC), phosphatidic acid (PA), monogalactosylmonoacylglycerol (MGMG), monogalactosylacylglycerols (MGDG), monoacylglycerol (MG), lysophosphatidylglycerol (LPG), lysophosphatidylethanolamine (LPE), lysophophatidylcholine (LPC), lysophosphatidic acid (LPA), digalactosylmonoacylglycerol (dgmg), digalactosyldiacylglycerol (DGDG), diacylglycerol (DG), coenzyme (Co), cardiolipin (CL), ceramide 1-phosphates (CerP), CerG3GNAc1, diglycosylceramide (CerG2), glucosylceramide (CerG1), ceramides (Cer), acylglucosyl-sitosterol esters (AGlcSiE)

To isolate exosome-like nanoparticles from *Panax ginseng*, raw fresh ginseng was thoroughly ground in 1X PBS. After sequential low-velocity centrifugation steps, large debris and impurities were removed from the ginseng juice. The sucrose cushion method was applied to prevent the aggregation and degradation of the exosomes, achieved by increasing centrifugal force (Fig. 1a, b). GENs were observed through sucrose gradient fractionation at 1.13–1.19 g/mL (Fig. 1c) and identified based on the average size of 151.6 nm with a low polydispersity index (PDI) ranges. The zeta potential of GENs indicated that GENs held a negative charge of –17.9 mV (Fig. 1d). The size distribution and particle concentration were measured by nanoparticle tracking analysis (NTA). Transmission electron microscopy (TEM) analysis indicated that the GENs were mostly spherical and uniform in shape, without aggregation or degradation. The quantification of GENs was performed by measuring the protein concentration using a Bradford assay kit, which indicated that GENs from ginseng were produced abundantly (approximately 1.68 mg per 10 g of ginseng tissue, based on the protein concentration). Those results suggest that homogenous GENs can be obtained on a large scale using a simple and eco-friendly method (Additional file 1: Fig. S1e).

To characterize the profile of GENs, we extracted total RNA, encapsulated lipids, and protein and performed RNA sequencing analysis, total lipid profiling, and proteomic analysis (Fig. 1e, Additional file 1: Table S2, S3). The results indicated that GENs mainly consisted of phospholipids enriched in phosphatidylcholine (PC; 28.8%), triglycerides (TG; 16.8%), and ceramides (Cer; 12.9%). A total of 98 miRNAs targeting various genes in C6 glioma cells and 86 proteins similar to those found in *Panax ginseng* were identified. We hypothesized that the abundant membrane lipids in GENs contribute to their stability and may facilitate preferential uptake by the BBB and brain tumor tissues.

In addition, the ginsenosides were extracted from GENs and analyzed through high performance liquid chromatography (HPLC). The extraction of ginsenosides from GENs was carried out under optimized extraction conditions. The main active components of ginsenosides, including Rg1, Re, Rg3, and Rb1, were detected in GENs at varying concentrations, which potentially contributes to its effectiveness in the treatment (Fig. 1f).

To assess the stability of GENs, their size and zeta potential were measured for 30 days. GENs were aliquoted and stored at –80 ℃. The size and zeta potential of GENs were measured to stay intact around 150 nm with a low PDI value, while the zeta potential of GENs maintained a negative charge of approximately –20 mV (Additional file 1: Fig. S1a, b). Typical freeze-drying of GENs was also performed to guarantee long-term stability. The morphology of GENs remained in the same uniform spherical shape after the freeze-drying as observed by TEM. The size and zeta potential of freeze-dried GENs showed slight increases compared to the fresh GENs (Additional file 1: Fig. S2a, b). Hence, the freeze-dried GENs were used in the consecutive experiments. To further analyze the stability and profile of GENs, the concentrations of total RNA and protein were measured. The integrity of total RNA extracted from ginseng tissue and GENs was analyzed by 10% denaturing polyacrylamide gel electrophoresis. It was observed that the total RNA of GENs was enriched, and the integrity and length of RNA fragments in GENs were slightly distinct from those extracted from ginseng tissues. As a result, the RNA fragments isolated from GENs consisted of various RNA fragments ranging in size from 50 to 500 base pairs (bp), while the RNA fragments isolated from ginseng tissues were 80–1000 bp in size.

In addition, the samples of total protein extracted from ginseng tissue and GENs were separated by 15% SDS-PAGE gel. The proteins isolated from ginseng tissues and those isolated from GENs revealed similar protein bands, indicating the reliable consistency of the protein components (Additional file 1: Fig. S2c–e).

### Effect of GENs on glioma cell apoptosis in vitro

**Fig. 2:**
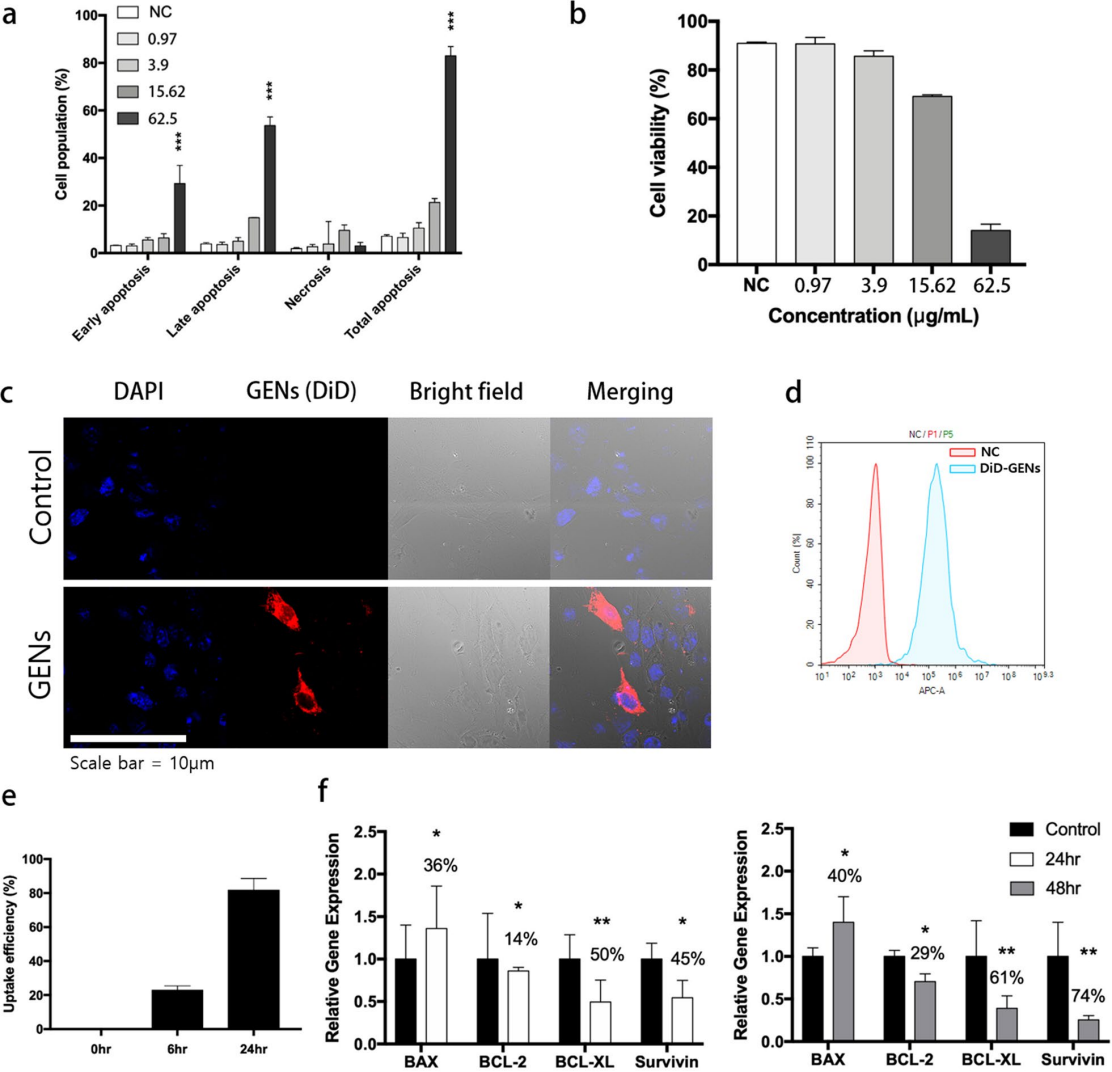
Apoptosis in C6 glioma cells treated with ginseng-derived exosome like nanoparticles (GENs) and cellular uptake of GENs. **a** Cell apoptosis assay in C6 glioma cells by Annexin V/PI after treatment with GENs at concentrations ranging from 0.975 to 62.5 μg/mL for 24 h (the concentrations were determined by Bradford assay). **b** Cell viability after treatment with GENs at the indicated concentrations was expressed as a percentage of cells in the control group (three independent experiments). **c** Confocal fluorescence imaging showed the internalization of conventional liposomes made from egg yolk and GENs by C6 glioma cells. After incubating C6 glioma cells with DiD-labeled GENs for 6 h, the distribution of GENs was assessed. Nucleic acid and GENs were stained with Hoechst (blue) and DiD staining dye (red), respectively. **d** Cellular uptake of GENs after 6 h of incubation. **e** Measurement of uptake efficiency of GENs in cells for 24 h. **f** Quantitative PCR analysis for the mRNA expression of apoptosis-associated genes in C6 glioma cells treated with GENs. 50 µg/mL of GENs was treated in C6 glioma cells for 24 h and 48 h. The mRNA expression levels were normalized to those of GAPDH (housekeeping gene) and expressed as a ratio of the control group (*, P < 0.05; **, P < 0.01; ***, P < 0.001)

To quantify the ratio of C6 glioma apoptosis, apoptosis induced by GENs was analyzed by an Annexin V/PI assay. As the concentration of GENs increased, the C6 glioma cells showed higher rates of early apoptosis (FITC + /PI−) and late apoptosis/necrosis (FITC + /PI +) compared with the negative control group (Fig. 2a, b). To study the effect of GENs on C6 glioma cell viability, the cells were treated with GENs at concentrations ranging from 1 to 62.5 μg/mL for 24 h. As a result, C6 glioma apoptosis was initiated by treatment of GENs at a concentration of 15.625 μg/mL and the total apoptosis was significantly increased at a concentration of 62.5 μg/mL of GENs, showing 81 ± 1.4% of apoptotic cell death. Collectively, flow cytometry analysis indicated that GENs could mainly affect C6 glioma cell apoptosis when administered at the highest concentration.

### Cellular uptake and internalization of GENs and in vitro BBB penetration

C6 glioma cells were treated with DiD-labeled GENs and analyzed by confocal microscopy to determine the internalization of GENs. As shown in Fig. 2c, after 6 h of incubation, the distribution of DiD-labeled GENs in cells significantly increased compared with DiD-labeled liposomes, indicating the selective uptake of GENs by C6 glioma cells. Also, the uptake of GENs continued to increase over a 24 h period (Fig. 2c–e). To stimulate the brain environment, an in vitro transwell BBB model was used (Additional file 1: Fig. S3). By utilizing a co-culture transwell membrane with a pore size of 0.4 μm, we aimed to assess the internalization of GENs through the BBB. Brain capillary endothelial cells (BCECs) were seeded on the upper compartment and incubated for 16 days until the transepithelial/endothelial electrical resistance (TEER) level reached above 200 Ω cm^2^ (Additional file 1: Fig. S3b). Prior to the 24 h incubation of GENs, C6 glioma cells were seeded in the lower compartment. Then, DiD-labeled GENs were placed in the upper compartment and further incubated for 12 h. C6 glioma cells in the lower compartment were collected and the efficient cellular uptake of GENs was observed. As a result, the DiD-labeled GENs could pass through and be taken up by C6 glioma cells (Additional file 1: Fig. S3c). Thus, GENs can be internalized into the tumor by penetrating through the mimicked BBB and have the potential for drug delivery to brain endothelial cells.

To evaluate the uptake mechanism of GENs into C6 glioma cells through the BBB, BCECs seeded on a transwell membrane were treated for 6 h with a series of inhibitors including chlorpromazine (an inhibitor of clathrin-mediated endocytosis), cytochalasin D (a phagocytosis inhibitor), methyl-β-cyclodextrin (lipid raft disruptor), colchicine (pinocytosis and phagocytosis inhibitor), and antibodies for occludin and claudin-5 (paracellular transport inhibitors) (Additional file 1: Fig. S4). Additionally, to evaluate the internalization of DiD-labeled GENs at low temperatures, BCECs on the transwell were incubated at 4 ℃ for 1 h, and then C6 glioma cells incubated with DiD-labeled GENs for 12 h in the upper compartment were collected, and the fluorescence intensity was measured (Additional file 1: Fig. S4). Compared with the control group, a significant reduction (60.1% and 60.2%) in cellular uptake was observed at 4 ℃ and with colchicin, a microtubule-disrupting drug. In contrast, chlorpromazine, methyl-β-cyclodextrin, and cytochalasin-D either had no affect or slightly disturbed the endocytosis of GENs by C6 glioma cells. Samples treated with occludin and claudin-5 antibodies, which block the transmembrane protein of tight junctions involved in paracellular transport, exhibited approximately 98.2% and 91.2% cellular uptake compared to the controls. The internalization of DiD-labeled GENs significantly decreased at low temperatures and upon the addition of colchicine. It can be concluded that low-temperature conditions can hinder the internalization of GENs, and that the cellular uptake mechanism of GENs in BCEC is mediated by phagocytosis.

To evaluate the cytotoxicity of GENs, an MTT assay was performed to assess the growth of C6 glioma cells after treatment with various concentrations of GENs (ranging from 0.97 to 31.25 μg/mL) (Additional file 1: Fig. S5). The proliferation of C6 cells was not decreased and remained at about 100% after treatment with 31.25 μg/mL GENs for 24 h, and the inhibitory concentration 50 (IC50) was determined to be 53 μg/mL (data not shown). These results indicated that GENs are safe and do not compromise cell viability. Furthermore, GENs could provide a safe and effective platform for anticancer therapy.

### Expression of apoptosis-related genes induced by GENs

To evaluate the effect of GENs on apoptosis in tumor cells, the induction of apoptosis was measured using western blotting and RT-qPCR. C6 glioma cells were incubated in the presence of GENs ranging from 0.24 to 62.5 μg/mL. Protein expression levels of BAX family genes were analyzed. As a result, the levels of the apoptosis-related gene BAX increased, whereas those of the anti-apoptotic genes such as BCL-2 and Survivin significantly decreased (Additional file 1: Fig. S6a). These observations indicate that GENs can activate BAX and downregulate BCL-2 and Survivin in C6 glioma cells in a dose-dependent manner (Additional file 1: Fig. S6b–g).

The results of RT-qPCR analysis showed consistent results with the western blotting findings. After 48 h of treatment with GENs, an upregulation of BAX mRNA was observed, whereas mRNA levels of BCL-2, BCL-xL, and Survivin were reduced by 29%, 61%, and 74%, respectively, compared with the control group (Fig. 2f)**.** Overall, GENs appear to induce apoptosis in brain tumor cells (Fig. 2f).

### GENs suppress cellular growth in glioma cells, fibroblasts, and endothelial cells

**Fig. 3:**
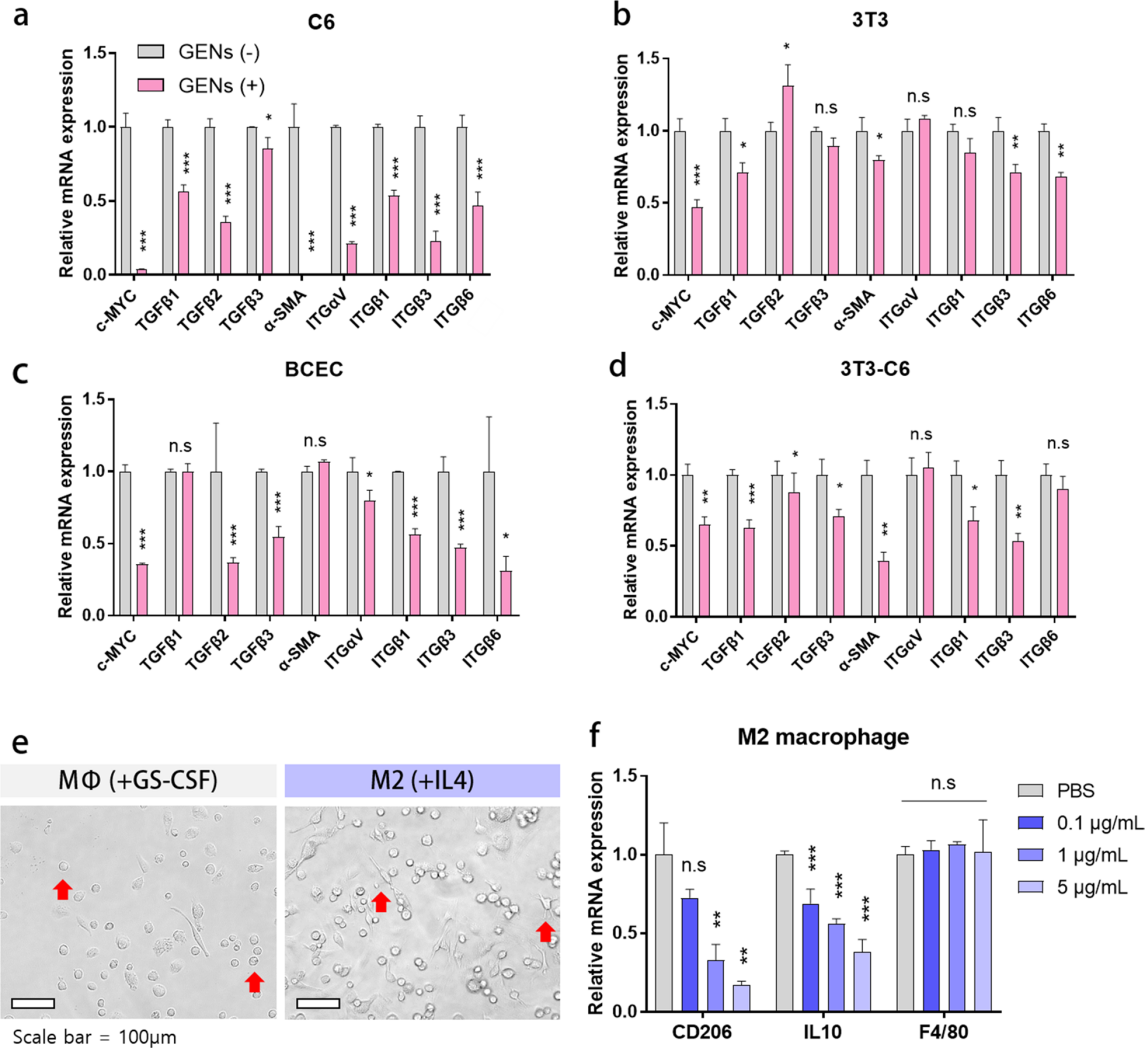
Inhibition of overexpressed c-MYC and activation of TGF-β and integrin in glioma cells, fibroblasts, and endothelial cells and the effect of treatment of M2 bone marrow-derived macrophages (BMMs). **a**–**d** Gene expression levels of c-MYC proto-oncogene, TGFβ1, 2, 3, integrin αVβ1, 3, 6, and α-SMA in C6, 3T3, BCEC, and 3T3-C6 co-culture. **e** The morphology of BMMs, as observed by microscopy. BMMs were stimulated with GS-CSF for 7 days and with IL4 for 3 days. **f** The mRNA expression levels in M2 macrophages after treatment with serially diluted GENs (*, P < 0.05; ** P < 0.01; ***, P < 0.001)

As shown in Fig. 3, we performed multiplex cytokine analysis to further assess the regulation of CAFs by GENs. We evaluated the mRNA expression levels of α-SMA, a marker of CAFs, and those of cell growth factors including c-MYC, TGF-β1/2/3, and integrin αVβ1/3/6 by RT-qPCR. After treatment with 10 μL of 1 mg/mL GENs for 48 h, we observed a significant decrease in the mRNA levels of c-MYC, TGF-β1/2/3, integrin αVβ1/3/6, and α-SMA. Similar trends were observed in cell growth factors in 3T3 cells and BCECs. However, the expression levels of some cytokines, such as TGF-β3 and integrin αVβ1 in 3T3 cells and TGF-β1 and α-SMA (a marker of CAFs) in BCECs, did not differ significantly.

Moreover, we co-cultured 3T3 and C6 at a 1:1 ratio to understand the role of GENs in mimicking TMEs. After treating the co-cultured cells with GENs at the same concentration as in the previous experiment, most cytokine genes, except those encoding integrin αV and integrin β6, were significantly downregulated (Fig. 3d). Collectively, GENs generally effectively suppressed cell growth factors, CAFs, and a marker of CAFs in vitro.

### GENs suppress M2 macrophage polarization

Macrophage phenotype transition is considered to be one of the major factors in developing an effective treatment option for tumors. To understand the effect of GENs on macrophages, we treated BMMs with GENs in vitro and assessed M2 macrophage polarization. Figure 3e shows the morphological differences in BMMs treated with various stimuli, including GS-CSF and IL4. Unstimulated macrophages, MФs, showed a relatively smaller and round morphology, while M2-treated macrophages showed a flat, elongated, and branching morphology. After treating the BMMs with serially diluted GENs ranging from 0.1 to 5 μg/mL, we evaluated the gene expression levels of CD206 and IL10, indicators of M2 macrophage polarization. As shown in Fig. 3f, compared with the control group, the mRNA expression levels of CD206 and IL10 in the treatment groups decreased with increasing concentrations of GENs. These results indicate that GENs effectively down-regulate M2 macrophage polarization and the expression of pro-tumoral cytokine without causing cell damage.

### Targeting effect of GENs in vivo

**Fig. 4:**
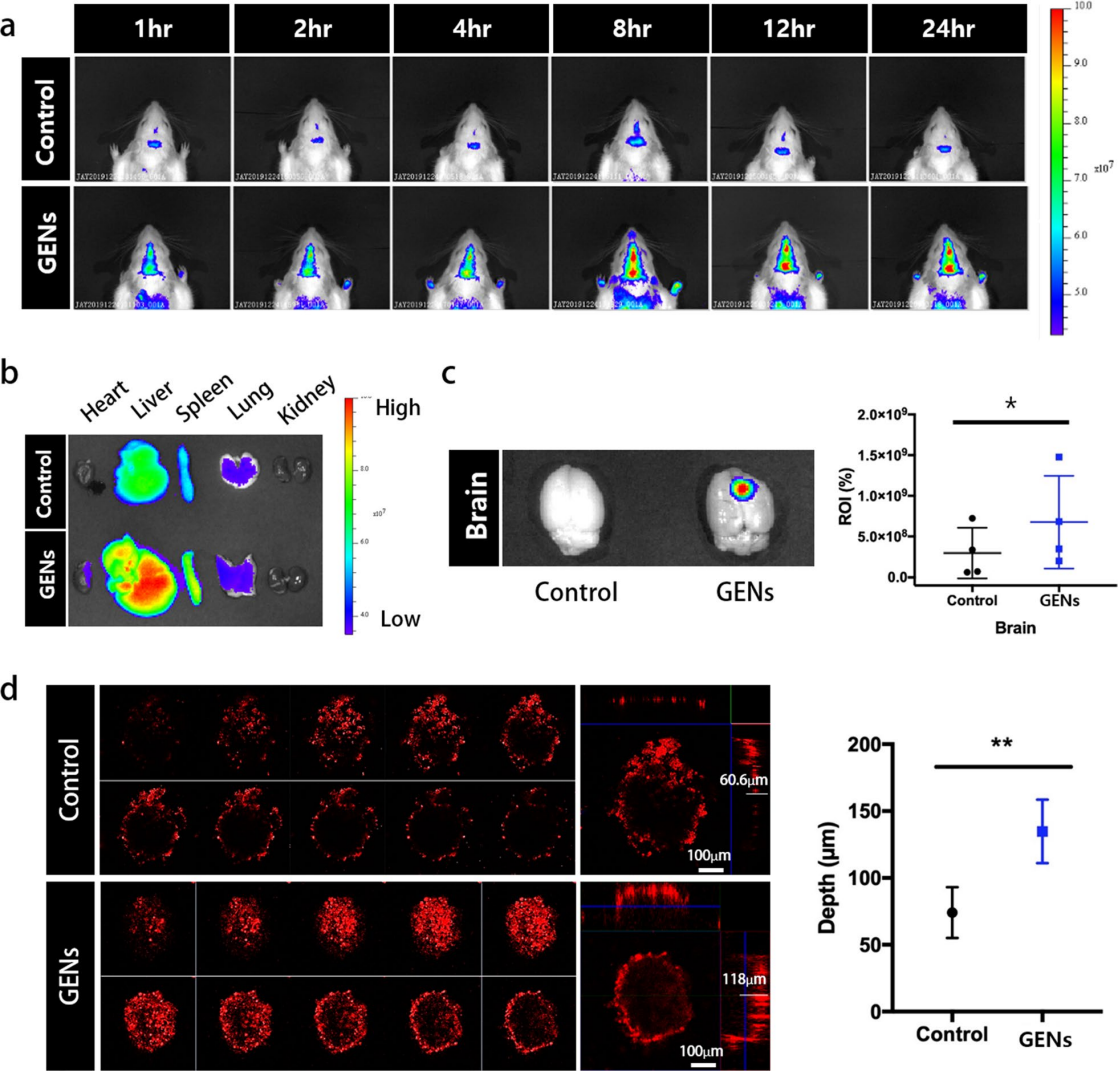
The targeting ability of GENs to glioma in vivo*.* **a** In vivo fluorescence imaging of DiD-labeled GENs. Conventional liposomes based on egg yolk were used as a control. After intravenous (IV) administration of DiD-labeled GENs, fluorescence imaging was performed at different time points. **b** Ex vivo fluorescence imaging of the dissected organs 24 h after the IV injection of DiD-labeled GENs. Higher fluorescence intensity in the brain was observed in GENs compared to the control group. **c** Relative region of interest (ROI) of fluorescence intensity in the brain samples. Using ROI as a measure, the graph shows that GENs exhibited an almost 2.26-fold higher fluorescence intensity on average than the control group. **d** Penetration of GENs and conventional liposomes into 3D tumor spheroids (*P < 0.05; **P < 0.01)

To evaluate the targeting ability of GENs in the brain tumor, we prepared 6–8-week-old Balb/C mice (18–20 g). DiD-labeled GENs were intravenously injected, and the fluorescence intensity of DiD-labeled GENs was measured using an in vivo imaging system (IVIS) at different time points over a time period of 24 h (Fig. 4a). Prior to each sample injection, we confirmed the comparable fluorescence intensity of conventional liposomes based on egg-yolks and GENs to ensure consistent experimental conditions (not shown). Compared with the conventional liposomes, GENs showed a rapidly increasing accumulation in the brain at all timepoints. In particular, GENs efficiently crossed the BBB and reached the tumor within 1 h after the administration. In addition, to determine the targeting effect and bio-distribution of DiD-labeled GENs, we dissected and imaged several organs including the heart, liver, pancreas, lung, kidney, and brain after 24 h of the injection of DiD-labeled conventional liposomes and GENs (Fig. 4a–c). GENs demonstrated a preferential targeting of brain tumors, showing a higher fluorescence intensity than conventional liposomes. By measuring the relative fluorescence intensities of the conventional liposomes and GENs in the brain by region of interest (ROI), we found that GENs resulted in almost 2.26-fold higher fluorescence intensity compared with the control group (Fig. 4c). We also observed a greater accumulation of GENs in the liver, spleen, lung, and partly in the heart compared to the conventional liposomes. These findings can be attributed to the physiochemical properties of GENs.

To further evaluate the selective and specific penetration ability of GENs in the steric tumor environment, we generated a mimic tumor model using cellular aggregates through 3D non-scaffold-based cell cultures (Fig. 4d). 10 days after seeding C6 glioma cells, the diameter of the spheroids was measured to be approximately 400 μm. DiD-labeled GENs and conventional liposomes were incubated with the tumor spheroids for 12 h. DiD-labeled GENs showed a much deeper penetration (118 ± 16.805 μm) compared with conventional liposomes (60 ± 13.445 μm) (Fig. 4d). We hypothesize that GENs have an advantage in infiltrating tumors through macropinocytosis and due to the overexpression of glucose transporters 1 (GLUT-1) in C6 glioma cells [1, 16, 17]. In support of this idea, when C6 glioma cells were pre-treated with Nocodazole and a GLUT-1 inhibitor, the cellular uptake of GENs was significantly inhibited (Additional file 1: Fig. S7). This suggested that macropinocytosis and the GLUT-1 pathway are the major pathways involved in the endocytosis of GENs in C6 glioma cells. Therefore, GENs demonstrate suitable physiological and chemical properties that enable enhanced intratumoral accumulation and fast penetration of the BBB.

### Antitumor effect induced by GENs in vivo

**Fig. 5:**
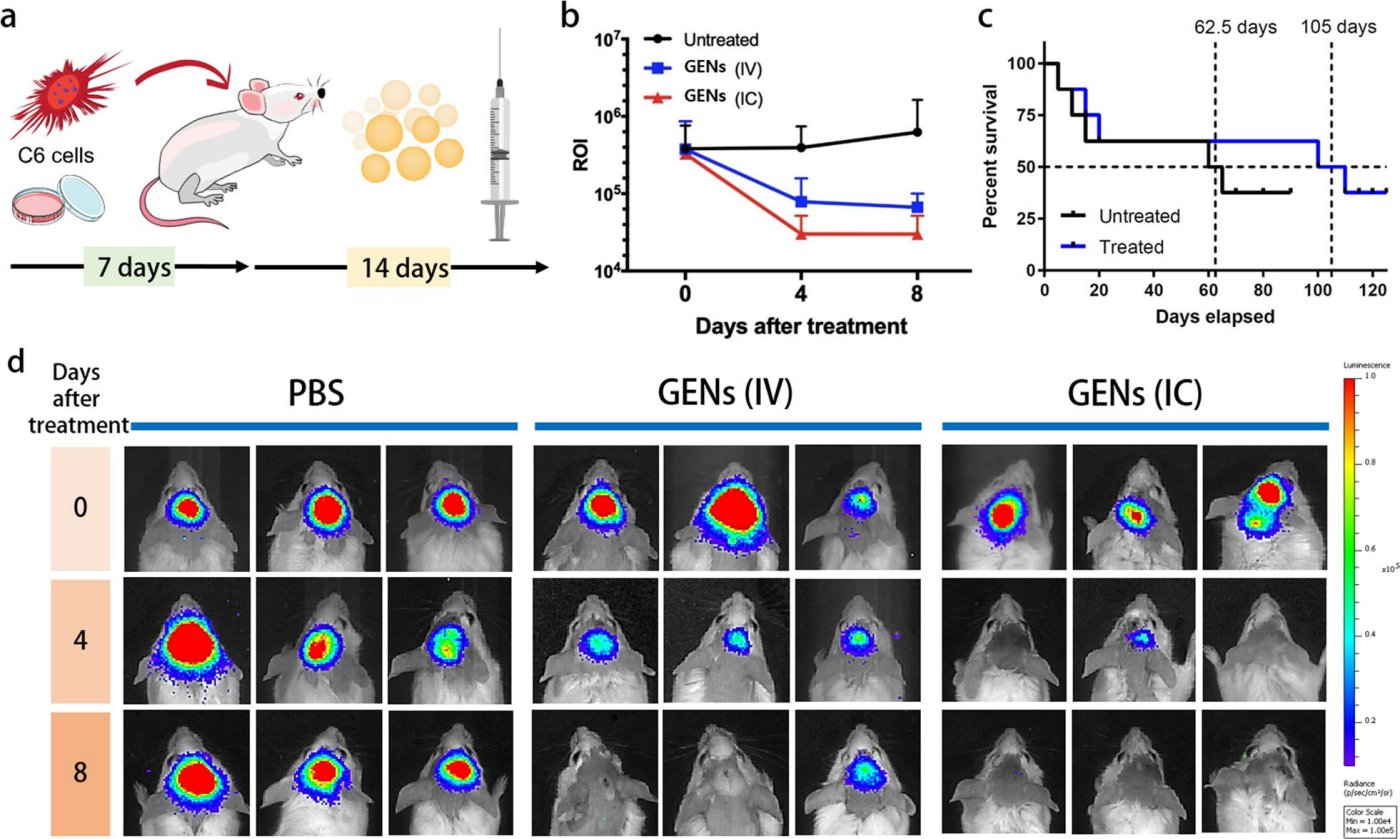
In vivo anticancer effect induced by GENs and Kaplan–Meier survival analysis. **a** Schematic illustration of the orthotopic model using a Balb/C mice. After the implantation of C6 glioma cells into the brain, GENs were injected every other day for 14 days. **b** A region of interest (ROI) of luminescence intensity compared to the control group after the indicated number of days after the treatment (0 to 8 days). **c** The survival rate of tumor-bearing mice treated with GENs compared to the control group (N = 8). **d** After orthotopic treatment, the luminescence intensity of C6 cells (expressing luciferase) was measured by an in vivo imaging system. On day 8 after the treatment, a significant reduction in the luminescence intensity of C6 glioma was observed in the group treated with GENs compared to the control group (N = 8)

To evaluate the anticancer effect of GENs, tumor-bearing Balb/C mice were treated with 5 μL of 2 mg/mL GENs (based on the protein concentration) through intracranial (IC) injection and 200 μL of 2 mg/mL GENs (based on the protein concentration) through intravenous (IV) injection every other day for 14 days after orthotopic tumor implantation (Fig. 5a). The progression of tumor growth was evaluated using an IVIS from day 0 to 8, and the luminescence intensities of tumors in the experimental groups were measured after the first injection of GENs (Fig. 5b). Figure 5b displayed the luminescence intensity of the tumor in a Balb/c mice, showing a higher luminescence intensity in the control group, indicating tumor growth, compared with the GENs’ treatment group. In mice receiving both IV and IC injections of GENs, the luminescence intensities of C6-Luc decreased markedly as the treatment continued. Notably, a significant decrease in the luminescence intensity of C6-Luc in the two experimental groups, especially in mice receiving IC injection within day 4 (Fig. 5b). The administration route appeared influence the efficacy of GENs, as they exhibited faster tumor reduction. The median survival time of the mice in the IV injection group was 105 days, significantly longer than that in the control group, with a median survival rate of 62.5 days (Fig. 5c).

**Fig. 6:**
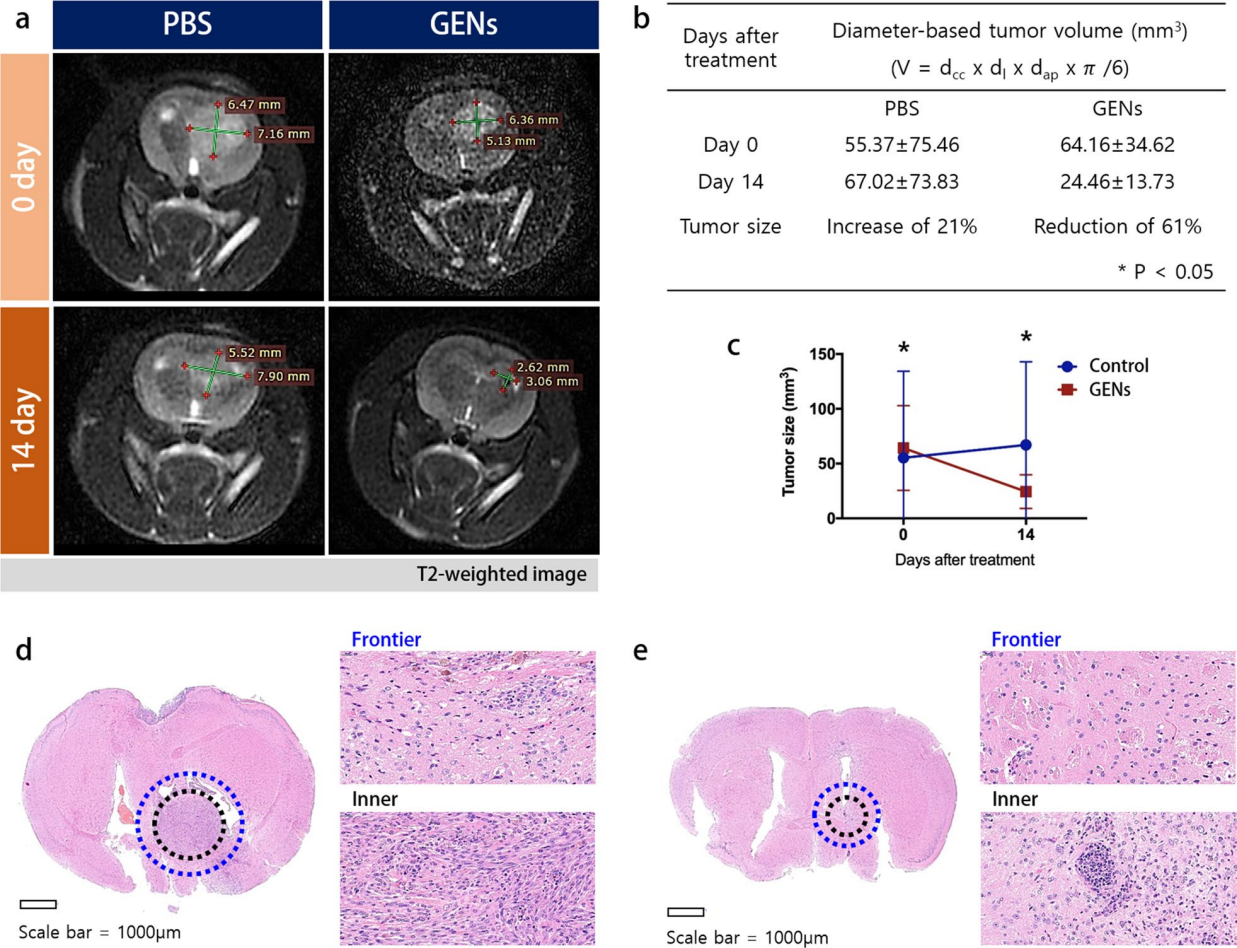
Orthotopic-induced xenograft brain tumor tissue hematoxylin and eosin (H&E) staining and magnetic resonance imaging (MRI) measurement of the C6 glioma in Wistar rat. **a** After 14 days of treatment with GENs, the tumor volume was measured by MRI imaging. **b**, **c** A table and graph for the diameter-based tumor volume. Tumors decreased in volume by 61% at day 14 after treatment with GENs, whereas tumors increased volume by 21% in the control group. **d**–**e** H&E staining of the brain tissue of an control tumor and treated tumor, respectively (*P < 0.05)

To visualize changes in the tumor mass within the brain, we utilized the Wistar rat, and measured the tumor volume of C6 glioma before and after treatment with GENs. Orthotopic implantation of C6 glioma cells was performed in 8-week-old Wistar rats (200–250 g). 14 days after the injection of GENs, tumor growth was monitored using magnetic resonance imaging (MRI) with T2-weighted images 14 days after the injection of GENs (Fig. 6a). As shown in Fig. 6b, tumor volume in the treatment group decreased significantly from 64.16 ± 34.62 mm^3^ to 24.46 ± 13.73 mm^3^, displaying a marked contrast with the control group where tumor volume increased from 55.37 ± 75.46 mm^3^ to 67.02 ± 73.8 mm^3^.

In addition, three mice from each group (control, IV, and IC) were sacrificed to collect brain tissues for analysis. The brain tissues were stained with hematoxylin and eosin (H&E) and cut in the coronal plane. The results revealed that a significant decreased in tumor size in the treatment groups, whereas an increase in tumor size was observed in the control group (Fig. 6d, e). The H&E-stained brain tumor biopsy treated by GENs showed a reduced and small population of tumor cells, while the control group displayed significant histologic atypical changes and a large population of tumor cell, as indicated by strong nuclear staining.

### Evaluation of liver toxicity of GENs in vivo

We further analyzed the cytotoxicity of GENs in vivo. (Additional file 1: Fig. S8) After 7 times of intravenous injections of GENs at the same concentration of treatment, the blood plasma was collected and the blood chemistry analysis including the blood aspartate aminotransferase (AST), alanine aminotransferase (ALT), albumin (ALB), alkaline phosphatase (ALP), urea, creatine, and uric acid was performed. As a result, those indicators of liver function were not elevated, indicating that the GENs were not harmful and did not induce toxicity in the liver.

### The regulation of CAFs leads to tumor-associated macrophages (TAMs)

**Fig. 7:**
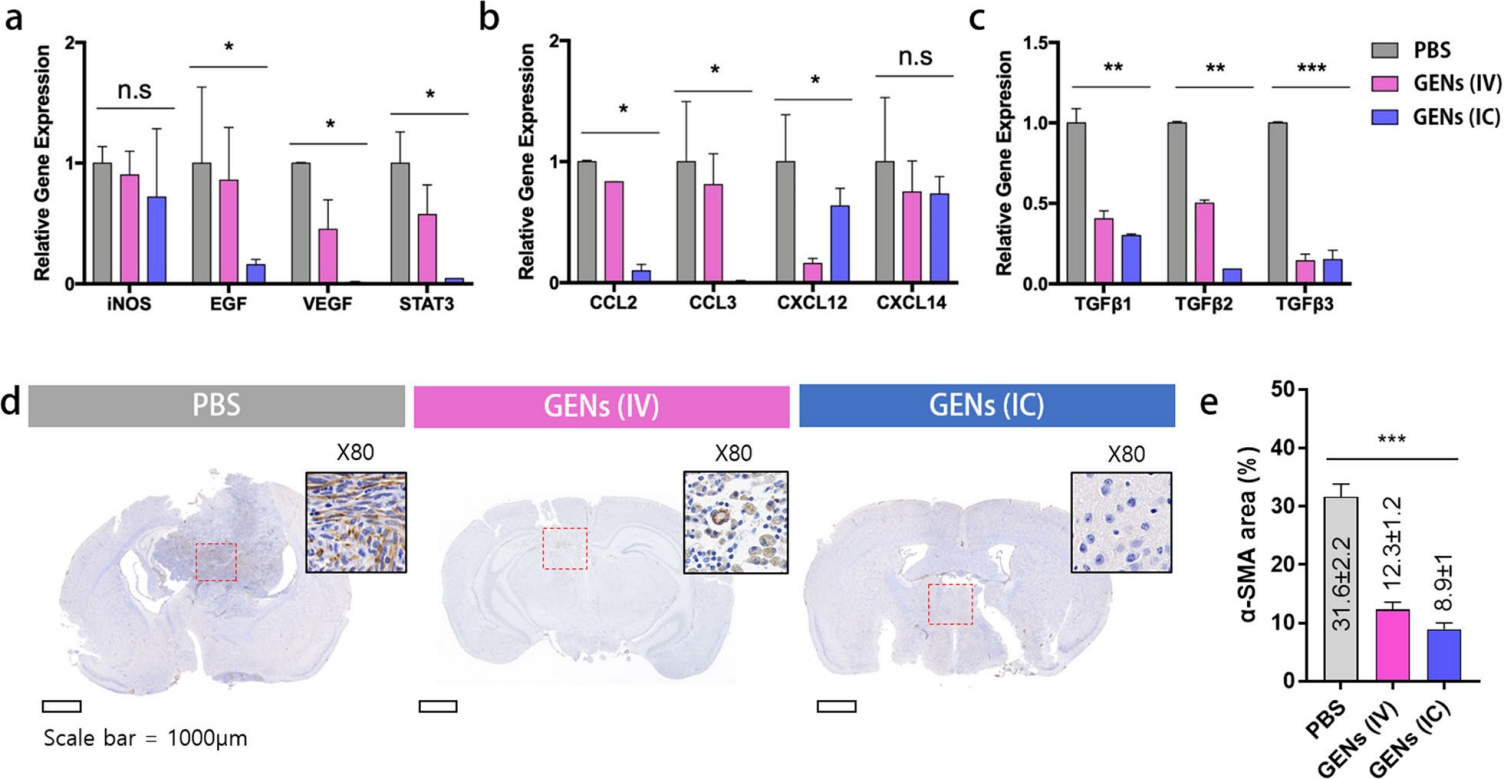
Gene expression of CAFs in the tumor microenvironment in tumor-bearing mice. **a** mRNA expression of CAF-related genes was decreased in the treatment group. **b** Reduced gene expression of chemokines. **c** Decreased gene expression of the TGFβ family in the treatment groups. Grey, PBS; pink, intravenous (IV) administration; blue, intracranial (IC) administration. **d**–**e** After treatment with GENs, α-SMA immunohistochemical staining in the brain tissue was determined (brown) (*, P < 0.05; **, P < 0.01; ***, P < 0.001)

Regulating the tumor microenvironment (TME) is considered to be an important approach for effective therapy. The TME consists of a heterogeneous population of cells, including infiltrating cancer cells, epithelial cells, endothelial cells, mesenchymal macrophages, and fibroblasts. Cytokines and chemokines are excessively secreted from various cells, and uncontrolled cell signaling induces oncogenesis and results in tumor progression. Thus, when evaluating the therapeutic effects against tumors, it is essential to consider various aspects. To validate the immunomodulatory effect of GENs, we first examined the gene expression levels of CAF in the TME in the mice. We assessed the mRNA expression levels of growth factors, angiogenic factors, and chemokines. Compared with the control group, we noted decreased mRNA levels of inducible nitric oxide synthase (iNOS), epidermal growth factor (EGF), vascular endothelial growth factor (VEGF), signal transducer and activator of transcription-3 (STAT-3), and chemokines including CCL2, CCL3, CXCL12, CXCL14, and the transforming growth factor beta (TGF-β) family in the treatment groups (Fig. 7a–c). Decreased iNOS expression is responsible for lower macrophage toxicity and can therefore induce an immune response to treat cancer and suppress tumor growth [41]. However, there was no significant difference in the mRNA expression levels of iNOS between the treatment groups. Interestingly, the expression levels of STAT3, which can potentially control cell growth and act as an oncogenic driver by regulating several apoptosis-related pathways, were significantly decreased in the treatment groups [6].

Additionally, the mRNA levels of chemokines CCL2, CCL3, CXCL12, and CXCL14 were also obviously reduced in the treatment groups. All things considered, GENs might not only be a regulator of macrophage proliferation but also contribute to the downstream signaling pathway of apoptosis-related signal molecules in TAMs. Thus, GENs have the potential to become a great adjuvant therapeutic strategy by reducing the expression of cytokines and promoting tumor suppression.

To investigate the role of GENs in CAFs, the in vivo expression of α-SMA in the brain tissue was analyzed by immunohistochemical staining (Fig. 7d, e). Immunohistochemical staining of the brain tissue treated with GENs showed that the expression of α-SMA was significantly reduced compared to the brain tissue treated with PBS. The ratio of α-SMA-positive area was 31.6 ± 2.2% in the control group, whereas it was reduced to 12.3 ± 1.2%, and 8.9 ± 1%, in the GENs IV injection and GENs IC injection group, respectively. These results confirmed that treatment with GENs effectively suppresses CAFs in the brain tumor-bearing mice, suggesting that GENs can serve as a promising therapeutic approach to enhance the effectiveness of cancer treatment and improve prognosis.

**Fig. 8:**
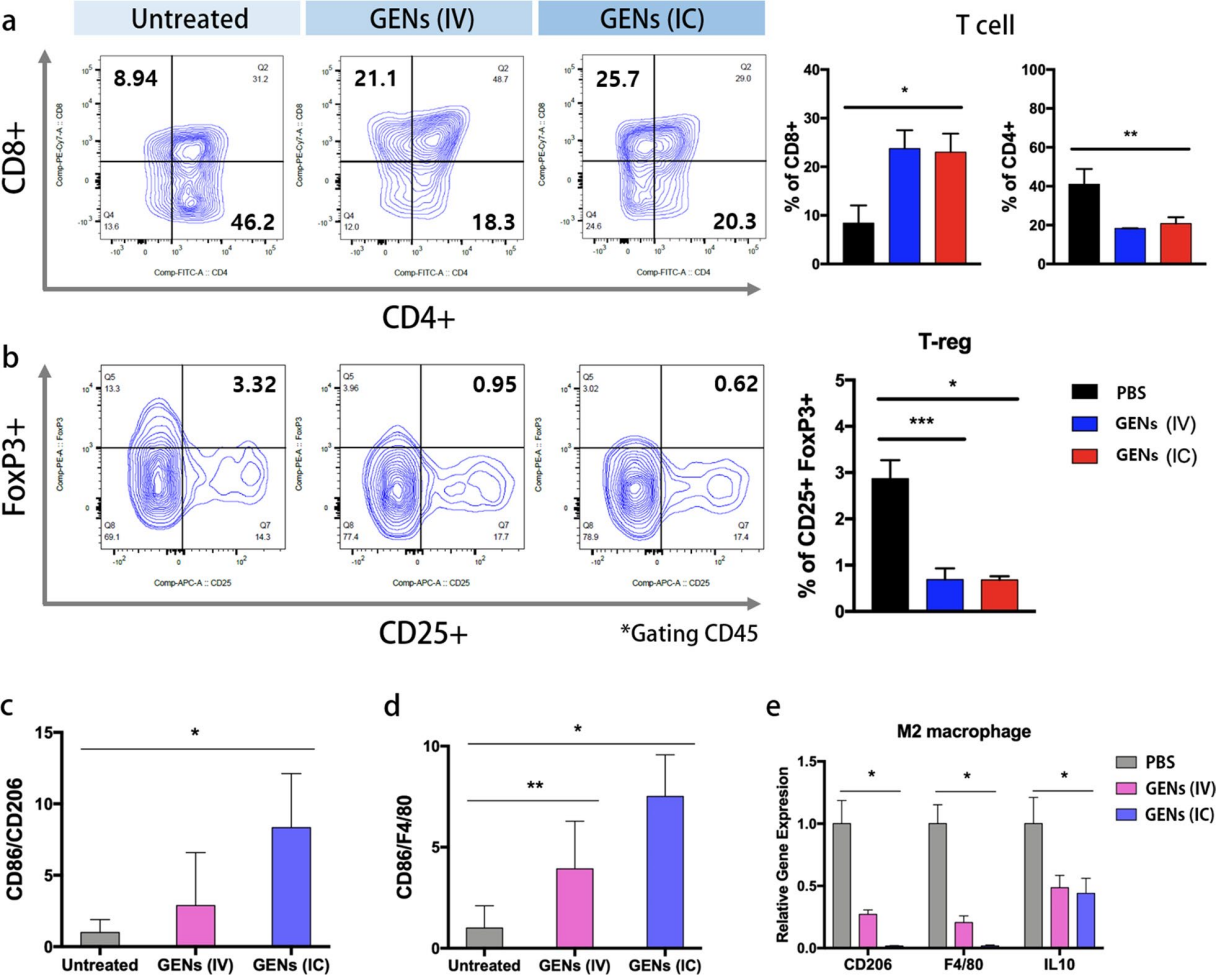
T cells and regulatory T cells (Tregs) are induced by decreased cytokines in the tumor microenvironment (TME). **a** Analysis of regulation of T cells. Fluorescently conjugated anti-CD8, CD4, and CD45 monoclonal antibodies were used to analyze T cells. The abundance of CD8 of T cells, gated CD45, was increased in the GENs treatment groups, whereas the abundance of CD4 was decreased compared to the PBS group. **b** Fluorescently conjugated anti-FoxP3, CD25, and CD45 monoclonal antibodies were used to analyze Tregs. Tregs cell population was evaluated by FACS analysis. The abundance of Tregs was reduced in the GENs treatment groups. **c**–**e** The ratio of the relative gene expressions of M1 and M2 (*, P < 0.05; **, P < 0.01; ***, P < 0.001)

We had a particular interest in understanding the regulation of macrophages by GENs in the TME. To gain further insights into immune regulation, we investigated the effects of GENs on T cells and regulatory T cells (Tregs) (Fig. 8). T cells plays a crucial role in defending against infections and cancer, while Tregs regulate the immune system. There is a close interaction between T cells and Tregs through several mechanisms. For example, Tregs can inhibit T cell function by secreting suppressive cytokines such as TGF-β, IL-10, and IL-35. In addition, Tregs can suppress anticancer immunity and impede immune surveillance, thereby promoting tumor growth and progression [42]. Consequently, targeting Tregs holds promise as an approach for cancer therapy. As shown in Fig. 8, treatment with GENs, led to a significant increase in CD8 + T cells, known as cytotoxic T cells, within the TME compared with the control group, whereas CD4 + T cells, known as helper T cells, were decreased (Fig. 8a). The abundance of Tregs (FoxP3 + and CD25 +) was notably reduced in the treatment groups (Fig. 8b). These findings support the previously described regulatory mechanism between Tregs and T cells. Molecular analysis of macrophages displayed in Fig. 8c–e led us to expect greater therapeutic effects on T cells and Tregs in response to GENs treatment through IC injection. However, the groups receiving GENs via IV and IC injections showed similar abundances of T cells and Tregs. We speculated that, assessing the proliferation of T cells and Tregs would require, more than one week after the treatment to observe a discernible difference in activation. The results may also be attributed to the attenuation of exponential regulation of accumulation after treatment. Immune cells were stimulated with appropriately concentrated GENs, and the immune cell analysis results were consistent with their maximal response to antigens. Previous studies have demonstrated that the expression of CD8 + and CD4 + in T cells is not significantly altered by different administration routes when a high concentration of vaccine is administered [43, 44]. Therefore, although different administration routes were employed, GENs were administered at a high concentration to all tumor treatment groups, likely resulting in the immune cells reaching a plateau in terms of their final expression. This hypothesis is supported by previous reports [43, 45].

To assess the impact of GENs on TAMs and their cytokine release, we conducted molecular analysis using M1 markers (including CD86), M2 markers (including CD206), and a pan macrophage marker (F4/80). M1-like macrophages are known to promote an antitumor response in TAMs by supporting a type 1 T helper cell (Th1)-mediated anticancer response and facilitating the recruitment of cytotoxic T cells. Based on the M1/M2 ratio, the treatment groups exhibited a general increase in M1-macrophages compared with the control groups, indicating an adjuvant antitumor effect on the glioma (Fig. 8c–e). The mRNA expression levels of M2-macrophage markers, CD206 and IL10, were significantly decreased in the treatment groups (Fig. 8e). M1 macrophages were induced with RAW 264.7 cells by adding 100 ng/mL lipopolysaccharide and 50 ng/mL interferon gamma (IFNγ). Subsequently these M1 macrophages were treated with GENs in vitro. The results showed a significant increase in CD86 expression, a marker of M1 macrophages, at all concentrations of GENs (Additional file 1: Fig. S9). Overall, in the GENs treatment groups, tumor suppression was observed through the promotion of macrophage proliferation and modulation of the immune system, including T cells and Tregs, which are involved in angiogenesis regulation.

### Gene silencing effect of ptc-miR396f on c-MYC

Previous studies reported [46, 47] the presence of bioactive compounds, including nucleic acids, in vegetables, which have shown potential in the treatment. Consequently, PENs containing genes could serve as a therapeutic RNA delivery agents themselves, benefiting from their inherent abundance of genes and biocompatibility. Furthermore, several specific genes including siRNAs and miRNAs derived from plants or vegetables have been functionally validated and offer new prospects for nucleic acid-based therapeutics targeting cancer and inflammation [46]. In addition, Teng et al. [47] reported that ginger-derived exosome-like nanoparticles that are preferentially taken up by gut biota, exerting regulatory actions on bacterial mRNA through their miRNAs. It is confirmed that the miRNAs within these nanoparticles can induce the production of IL-22, which is associated with the improvement of intestinal barrier function. However, it is not clear whether the miRNAs in GENs play a role in suppressing tumor growth and inducing antitumor immunity in cells and the TMEs.

**Fig. 9:**
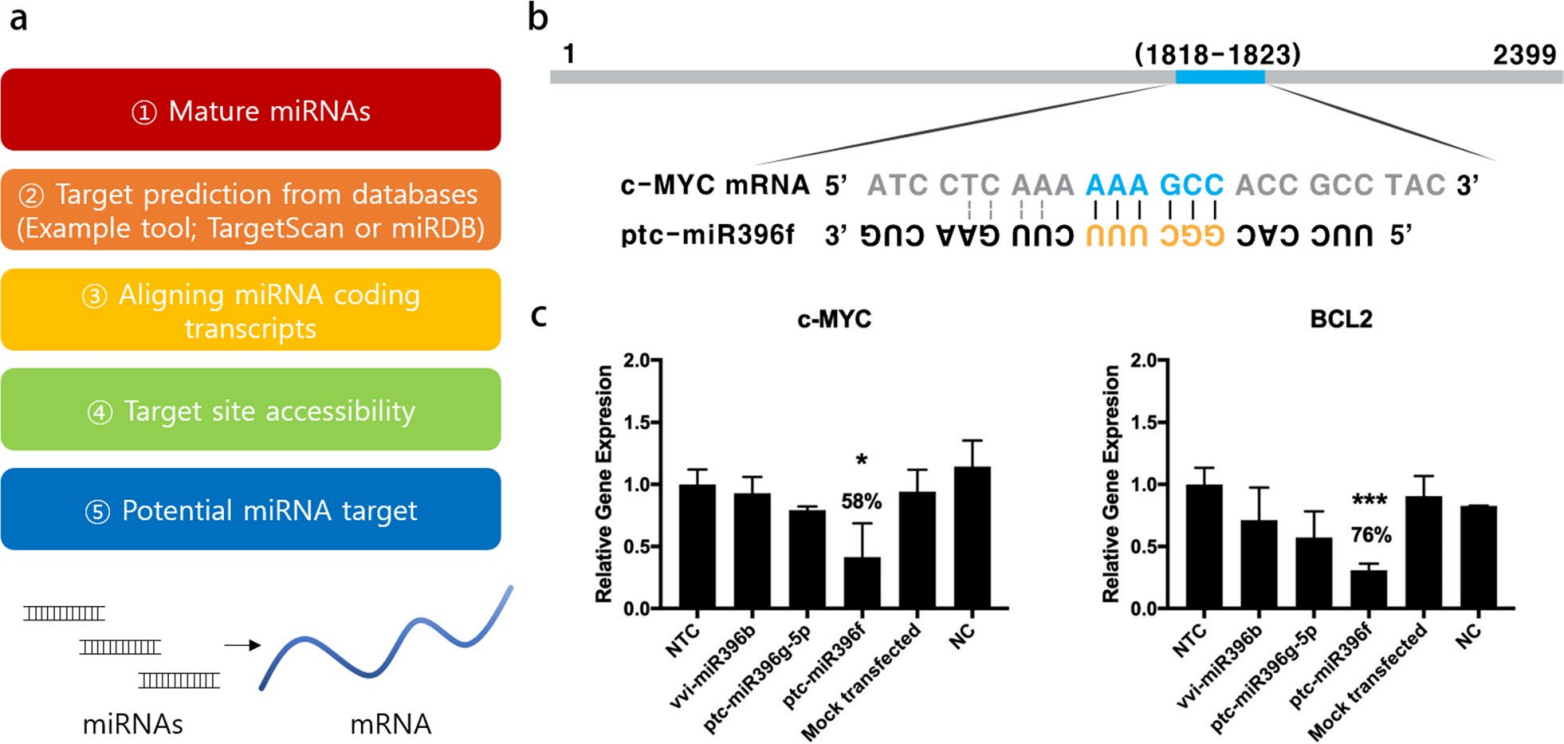
Prediction of miRNA targets and gene silencing effect of miRNAs mediated apoptosis of tumor cells through the regulation of apoptotic c-MYC signaling pathway. **a** miRNA targets were predicted using miRNA target prediction tool based on sequence-based miRNA target prediction. **b** The binding site of ptc-miR396f in the mRNA of c-MYC. **c** Relative gene expression levels of c-MYC and BCL2 with miRNAs. There was a significant gene silencing effect compared to mock-transfected and control groups, which are normalized to non-targeting control (NTC) (*, P < 0.05; ***, P < 0.001)

Hypothesizing that miRNA might be a regulatory factor, we analyzed the miRNA profile of GENs. Subsequently, we utilized the miRNA databases (TargetScan and miRanda) to identify miRNAs in GENs and predicted their potential targets based on sequence-based miRNA target prediction (Fig. 9a). miRNA targets were sorted by cumulative weighted context +  + score. Among the miRNA targets, we selected the target gene and potential oncogene and further examined the target site accessibility of miRNAs in the coding transcripts [48, 49]. Then, a potential miRNA-target interaction was identified. c-MYC is a predominant oncogene and the c-MYC signaling pathway is one of the well-known pleiotropic gene-regulating signal pathways, including BCL2 and TGF-β (Fig. 9b) [50, 51]. In C6 glioma cells, a parallel reduction was observed in gene expression levels of c-MYC and BCL2 after transfection with the miRNAs named vvi-miR396b, ptc-miR396f, and ptc-miR396f, compared to non-targeting controls (Fig. 9c; Additional file 1: Tables S1–S5). The highest gene silencing effect was seen with ptc-miR396f in both target genes, with 58% and 76% reductions in c-MYC and BCL2. In addition, to make the result of RNA sequencing data more helpful and clear, a pie chart of the miRNA abundance by miRNA family and the ten most abundant miRNAs in GENs by the read count were presented in Additional file 1: Fig. S10 and Fig. S11.

Given that the lipid composition of GENs primarily consists of phospholipids (28.8% of PC), we anticipated that we could demonstrate the efficient delivery of miRNAs using the lipid component of GENs as a delivery vector [47]. To examine whether the suppression of gene expression was induced as expected, the miRNAs were delivered via GENs. We extracted lipids from GENs using the Bligh and Dyer lipid extraction protocol and used the lipid content of GENs as a transfection reagent in conjunction with the miRNAs (Additional file 1: Fig. S12a). The delivery of ptc-miR396f via the extracted lipids exhibited the highest gene silencing effect on both c-MYC and BCL2 (Additional file 1: Fig. S12b).

In this study, we evaluated the anticancer activity and the regulatory effects of GENs on glioma. Various miRNAs involved in diverse signaling pathways related to apoptosis or cell proliferation were examined. The findings of this study confirmed that GENs are capable of suppressing tumor growth by inducing apoptosis in tumors, regulating macrophages and TAMs, and exerting gene silencing effects through exosomes. The results highlight the promising potential of GENs for the treatment of glioma.

### Gene Ontology (GO) classification

A total of 7,072,614 genes in GENs were determined by GO enrichment analysis. The results indicated that these genes were involved in biological processes (BP), cellular components (CC), and molecular functions (MF) (Additional file 1: Fig. S14). In the categories, the most abundant subcategories were cellular component (5427 genes) and organic substance metabolic process (5408 genes). The top 5 most prominent enrichment subcategories involved in the BP category were organic substance metabolic process (5408 genes), primary metabolic process (5204 genes), cellular metabolic process (5033 genes), DNA metabolic process (1039 genes), and organic acid metabolic process (803 genes).

### Scatter plot of differential gene Kyoto Encyclopedia of Genes and Genomes (KEGG) enrichment

To further understand the mechanism of the differential expression of genes (DEGs) from the GENs described above, we implemented KEGG pathway enrichment testing. The degree of KEGG enrichment was evaluated by the qvalue, the rich factor, and the number of genes enriched in the pathway [52]. The enrichment represents the ratio of the proportion of DEGs involved in the pathway to all genes. The higher rich factor is, the more significant the enrichment level of the DEGs in the pathway. The top 20 pathways with the most significant enrichment after screening are presented in Additional file 1: Fig. S15. These genes were enriched in the porphyrin and chlorophyll metabolism, propanoate metabolism, ABC transporters, alanine, aspartate, glutamate metabolism, and pyrimidine metabolism pathways.

## Discussion and conclusion

GENs have emerged as a promising anti-glioma drug option due to their significant therapeutic effects, demonstrated safety, and stability. Moreover, the simple isolation method allows for a high yield of GENs, making them a potential candidate for the development of nanodrugs and nanocarriers. GENs taken derived from *Panax ginseng* offer an excellent platform for drug delivery, as they contain various biologically active components including proteins, genes, lipids, and metabolites.

GENs have been proposed to be effective against various diseases including cancers, but only few studies have been conducted to support this idea. The findings of this study provide compelling evidence of the strong anticancer effects, especially on gliomas, by regulating cell proliferation and modulating the TME. As depicted in Fig. 7**, **Fig. 8**, **Fig. 9, we found that GENs possesses remarkable suppression of tumor growth suppression capabilities through regulatory mechanisms underlying their anti-cancer actions. The results indicate that GENs can serve multiple roles in targeting and eliminating cancers.

Exploring the internalization mechanism of GENs into targeted cells using inhibitors can increase our understanding of how GENs penetrate and transport their components into cells (Additional file 1: Fig. S4). Our data show that GENs predominantly utilize diverse endocytosis pathways for efficient uptake by targeted cells such as C6 glioma cells and BCECs. Even in a transwell model that stimulates the BBB or the blood–brain barrier tumor, GENs exhibit efficient penetration through closely spaced epithelial cells.

However, only limited information is available to display the activities and potential of GENs in the development of pharmaceuticals. As we presented in the supporting information, our team supports GENs to overcome problems with certain drugs, such as multiple drug resistance and metastasis.

In addition, we had to consider the inclusion of a control in the experiments. Therefore, we selected ginseng and ginseng extract (powder form) as the control groups to explore the effects of GENs. However, we encountered several experimental challenges with the control groups during the course of the study. First, it was difficult to quantify the concentration (or dose) of ginseng and ginseng extract in comparison to GENs. Ginseng and ginseng extract contain various components, both bioactive and inactive, such as carbohydrates, sugar, and fat, making it challenging to determine the proper dosage for glioma treatment solely based on total protein concentration. Second, ginseng and ginseng extract were not purified and could potentially contain contaminants. This made it problematic to directly conduct in vitro and in vivo experiments. Therefore, we were hesitant about using ginseng and ginseng extracts as controls, and we were not able to anticipate obtaining meaningful results with them. However, it is important to explore an appropriate control that is purified and enables accurate dosage analysis in future studies.

In conclusion, in terms of therapeutic applications, we view GENs as a potential nanomedicine with potential in cancer treatment. We anticipate that future studies will delve into the examination of GENs in combination with conventional drugs, explore other applications of GENs, and investigate possible modifications to enhance their targeting ability.

### Supplementary Information

**Additional file 1: Fig. S1.** Stability and characterization of GENs. **Fig. S2.** Characterization of GENs. **Fig. S3.** Transwell of C6 glioma cells and BCECs. **Fig. S4.** Blocking BBB endocytosis of DiD-labeled GENs in C6 glioma cells in vitro.* Fig. S5.* Cell viability of GENs was individually evaluated by MTT assay in C6 glioma, 3T3, RAW 264.7, and HUVEC. **Fig. S6.** Western blotting of protein expression of apoptosis-related proteins with sequential reduction of GENs’ concentrations from 62.5 to 0.24 μg/mL. **Fig. S7.** Inhibition of endocytosis of GENs with chemical inhibitors in C6 glioma cell. **Fig. S8.** Blood chemistry analysis in serum in vivo. **Fig. S9.** Flow cytometry analysis of CD86 expression in RAW 264.7 with the treatment of different concentrations of GENs. **Fig. S10.** A pie chart of miRNA abundance in GENs. **Fig. S11.** A pie chart of the ten most abundant miRNAs in GENs. Fig. S12. Lipid extraction from GENs and the exosomal delivery of miRNAs. **Fig. S13.** Encapsulation of miRNA in GENs. **Fig. S14.** Enriched gene ontology (GO) terms (hsa). **Fig. S15. **Gene Kyoto Encyclopedia of Genes and Genomes (KEGG) enrichment analysis of GENs. **Table S1.** Primer set list. **Table S2.** RNA Profiling Analysis. **Table S3.** Protein profiles of GENs. **Table S4.** hsa.DEG_GO_enrichment_result. **Table S5.** Readcount_TPM

## Data Availability

The data and materials used in the study are available from the corresponding author.
