# Supplementary material for: Anti-glioma effect of ginseng-derived exosomes-like nanoparticles by active blood–brain-barrier penetration and tumor microenvironment modulation
Source: J Nanobiotechnology. 2023 Aug 4;21:253. doi: 10.1186/s12951-023-02006-x (PMC10401762; doi:10.1186/s12951-023-02006-x)
Supplement: Supplementary file 1 — Additional file 1: Fig. S1. Stability and characterization of GENs. Fig. S2. Characterization of GENs. Fig. S3. Transwell of C6 glioma cells and BCECs. Fig. S4. Blocking BBB endocytosis of DiD-labeled GENs in C6 glioma cells in vitro. Fig. S5. Cell viability of GENs was individually evaluated by MTT assay in C6 glioma, 3T3, RAW 264.7, and HUVEC. Fig. S6. Western blotting of protein expression of apoptosis-related proteins with sequential reduction of GENs’ concentrations from 62.5 to 0.24 μg/mL. Fig. S7. Inhibition of endocytosis of GENs with chemical inhibitors in C6 glioma cell. Fig. S8. Blood chemistry analysis in serum in vivo. Fig. S9. Flow cytometry analysis of CD86 expression in RAW 264.7 with the treatment of different concentrations of GENs. Fig. S10. A pie chart of miRNA abundance in GENs. Fig. S11. A pie chart of the ten most abundant miRNAs in GENs. Fig. S12. Lipid extraction from GENs and the exosomal delivery of miRNAs. Fig. S13. Encapsulation of miRNA in GENs. Fig. S14. Enriched gene ontology (GO) terms (hsa). Fig. S15. Gene Kyoto Encyclopedia of Genes and Genomes (KEGG) enrichment analysis of GENs. Table S1. Primer set list. Table S2. RNA Profiling Analysis. Table S3. Protein profiles of GENs. Table S4. hsa.DEG_GO_enrichment_result. Table S5. Readcount_TPM [file 12951_2023_2006_MOESM1_ESM.zip › 12951_2023_2006_MOESM1_ESM/Supporting information_Final_v9.1.docx]

**Additional file 1**

**Anti-glioma Effect of Ginseng-Derived Exosomes-like Nanoparticles by Active Blood-brain-barrier Penetration and Tumor Microenvironment Modulation**

*Jisu Kim**^1^, Ying Zhu**^1,^* *^2^, Sunhui Chen^3^, Dongdong Wang^4^, Shuya Zhang^1^, Jiaxuan Xia^1^, Shiyi Li^1^, Qiujun QIU^1^, Hyukjin Lee^5^, Jianxin Wang*^, 1,2^*

*^1^* *Department of Pharmaceutics, School of Pharmacy,* *Fudan University & Key Laboratory of Smart Drug Delivery, Ministry of Education, Shanghai 201203, People’s Republic of China*

*^2^* *Department of Oncology, Shanghai Medical College, Fudan University, Shanghai, 200032, People’s Republic of China*

*^3^ Department of Pharmacy, Fujian Provincial Hospital, Fuzhou, 350001, People’s Republic of China*

*^4^ Department of Radiology, Huashan Hospital, Fudan University, Shanghai, 200040, People’s Republic of China*

*^5^ College of Pharmacy, Graduate School of Pharmaceutical Sciences, Ewha Womans University, Seoul 03760, Republic of Korea*

**Materials and Method**

**Transmission electron microscopy (TEM)**

10 μL of 10 μg of GENs (based on the protein concentration of GENs) was spotted onto carbon-coated grids at room temperature for 10 min. The carbon-coated grids were immediately stained in 2% phosphotungstic acid at RT for 30 min. The nanoparticle imaging was conducted by transmission electron microscope (JEM-F200, JEOL, Japan)

**Nanoparticle tracking analysis (NTA)**

Nano particle size distribution and number concentration of GENs were determined using NTA (NanoSight LM10, Malvern Instrument, UK). Briefly, GENs were gently agitated and diluted to a final dilution of 1: 10,000. Subsequently, 1 mL of GENs was injected in the sample chamber with disposable sterile syringes (BD Discardit II, New Jersey, USA) until the solution flowed out of the nozzle. The sample was then analyzed for 60 sec using NanoSight automatic analysis settings at room temperature. The NanoSight NS300 software merged the average and SD values based on mathematical estimates derived from the sizes of all the particles analyzed by the software.

**HPLC analysis**

To isolate ginsenoside components from GENs, 0.5 g of GENs was dissolved in 1X PBS. Then, 600 μL of the clear phase of the organic solvent mixed with 1 mL of distilled water and Butanol (2:1, v/v) were mixed. After centrifugation at 5,000 g for 10 min, the upper phase of the sample was transferred to a fresh tube. The yellow solution was evaporated at 80°C for 10 min using a Rotary Vacuum Evaporator Water Bath to remove the organic solvent and the sample was dissolved by adding 100 μL of methanol.

When performing HPLC analysis, the ginsenoside components were eluted through the column of Agilent ZORBAX SB-Aq 5 μm, 4.6 Х 250 mm (CA, USA) at a flow rate of 1 ml/min and separated using a mobile-phase gradient. (Mobile phase A; distilled water, mobile phase B; acetonitrile) The analysis time was set for 80 min and the gradient was subsequently changed in mobile phase B to 19% at 0 min, 19% at 35 min, 29% at 55 min, 29% at 70 min, 31.6% at 77 min, 19% at 78 min, and ended at 80 min. The ginsenosides were analyzed using an Agilent 1260 Infinity II instrument. (CA, USA)

**Preparation of liposome using egg yolk**

The conventional liposome using egg yolk was prepared by the thin-film hydration method [1-3]. Briefly, 6 mg of cholesterol and 20 mg of EPC (w/w) were dissolved in 2 mL of ethanol/chloroform (1:1, v/v). The solution was placed into a round bottom flask and subjected to rotary evaporation using a ZX-98 rotary evaporator (LOOYE, China) for 10 min at 250 rpm, 48℃. The lipid film was evaporated to remove ethanol/chloroform traces. Then, the dried lipid film was dissolved with 5 mL of 1X PBS and incubated with gentle rotation for 30 min in a water bath at 48℃. During this process, homogeneous lipid suspensions containing different sizes of liposomes were generated. To homogenize the size distribution, the liposome suspensions were subjected to ultrasonic treatment in an ultrasonic cell pulverizer (200 W, on 5 s, interval 5 s, 40 cycles). The final lipid suspensions were stored at 4 °C for further utilization. DiD-labeled conventional liposomes were prepared by adding 4 μL of 1 mg/mL DiD-lipophilic dye prior to the evaporation step.

**Cell culture**

C6 glioma cells and BCECs were cultured in the same condition. Cells were in Dulbecco’s modified Eagle’s medium (DMEM) (Gibco BRL, Grand Island, NY, USA) with 10% fetal bovine serum (Hyclone, Logan,UT, USA), 1% penicillin, 1% streptomycin, and 1% MEM non-essential amino acid. The cells were incubated in a humidified atmosphere of 95% air and 5% CO_2_ at 37 °C.

**Isolation of total protein from GENs**

To visualize protein bands of ginseng and GENs by western blotting, total proteins were extracted from ginseng and GENs with medium RIPA lysis buffer (Yeasen, China) according to the manufacturer’s instructions. 150 μL of medium RIPA lysis buffer was added in 0.1 g of ginseng and 1 mL of GENs and centrifuged at 12,000g for 15 min at 4℃. The supernatant was collected and the protein concentration was determined using Bradford assay kit.

**Western blotting**

Total proteins were isolated from C6 glioma cells treated with GENs ranging from 62.5 µg/mL to 0.24 µg/mL for 48 h. According to a standard protocol of western blotting, the proteins were analyzed. To determine the protein concentration of C6 glioma cells treated with GENs, 2 × 10^5^ cells/mL were cultured in a 6-well plate and incubated for 48 h. The media was gently removed, washed with 1X PBS three times and thoroughly eliminated. 150 μL of RIPA lysis buffer with a protease inhibitor [25mM Tris-HCl (pH 7.6), 150mM NaCl, 1% NP-40, 1% sodium deoxycholate, 0.1% sodium dodecyl sulfate (SDS)] was added to each cell plates. The supernatant was collected and centrifuged at 12,000g for 15 min at 4℃. Protein concentration was determined using Bradford assay kit. For analysis, 20 mg of total protein was heated for 10 min at 100℃. Proteins were loaded on a 12% SDS-PAGE (SDS polyacrylamide gel electrophoresis) and transferred to polyvinylidene fluoride membranes (Nitrocellulose Membrane, 0.45 μm, Thermofisher, USA) with running electrophoresis at 200 volts for 40 min. The transferred membrane was blocked by 5% skim milk in PBS and immunostained with secondary antibodies.

***In vitro* uptake of GENs**

4 × 10^4^ cells/100 μL were deposited on the glass and DiD-labeled GENs were added. C6 cells were incubated for 6 h while avoiding lights. The glass was washed three times with PBS and then collected. The cellular uptake of GENs was observed using confocal microscopy at a wavelength (excitation/emission at 640 nm/680 nm). Images are representative of at least three independent experiments.

**Cellular uptake by flow cytometry**

To determine cellular uptake of GENs, C6 glioma cells (2 × 10^5^ cells/well) were incubated with 50 ng DiD-labeled GENs at 37℃ for 6 h. The media was eliminated and the cells were collected by trypsin treatment to detect the internalized GENs. Then, the cells were harvested and analyzed by flow cytometry. The result was organized by FlowJo software (BD Biosciences, USA)

**Internalization mechanism**

To explore the mechanism of cellular uptake of GENs in C6 glioma cells, inhibitors including 20 µM of chlorpromazine, 20 µM of Nystatin, 50 nM of Nocodazole, 20 µM of GLUT-1 inhibitor, 20 µM of methyl-β-cyclodextrin, 100 µM of sucrose were used.

**Preparation of transwell BBB model and cellular uptake**

In preparing a 6-well plate, 5 × 10^4^ cells/mL were seeded on the upper chamber of 0.4 μm of transmembrane. The cells were cultured for 14 days until the TEER reached over 200 Ω·cm2. Before the day of the experiment, 1 × 10^5^ of C6 glioma cells were seeded and incubated for 24 h. Subsequently, 10 µL of DiD-labeled GENs were added to the upper chamber of the transwell and incubated for 12 h.

**Penetration of the tumor spheroid model**

To demonstrate the miniature 3D tumor spheroids, 100mL of 2% agarose gel in cell culture media was sterilized at 120 ℃ for 30 min and the agarose gel was added to a 96-well plate. C6 glioma cells were seeded at a density of 1 x 10^4^ cells each well and cultured for 10 days until the size of spheroids grew up to 400 μm. The 3D tumor spheroids were incubated with the DiD-labeled GENs and conventional liposomes for 12 h. (N=10) Then, the 3D spheroids were taken out and washed with 1X PBS three times. The morphology and penetration of GENs were observed using confocal microscopy.

**Blood chemistry analysis**

0.5 mL of blood samples were taken from the mice through the retro-orbital method and was placed in tubes containing heparin (ﬁnal heparin concentration: 10 U/mL). The samples were centrifuged at 1000×g in 4°C for 10 min. The plasma was collected for the determination of liver function test. Aspartate aminotransferase (AST), alanine aminotransferase (ALT), albumin (ALB), alkaline phosphatase (ALP), urea, creatine, and uric acid values were measured using detection kits (Sigma Aldrich, USA) according to the manufacturer’s instructions.

**Quantification of bioluminescence imaging in vivo**

The bioluminescent intensity was assessed 5 min after 1mL of 15 mg of D-luciferin by IV injection. It was imaged with IVIS using the settings of exposure time 2-10 sec, large binning, F/Stop = 1.

**MRI imaging**

An MRI was performed on all rats to evaluate tumor invasion and growth in the brain. All rats were scanned with T2-weighted image mode set on 73.3 s of effective echo time (TE) and 7 of repetition time (TR) with slices 2 mm apart.

**Immunostaining by flow cytometry *in vivo***

Fresh tumor tissue was extracted from the tumor-bearing mice and harvested cells. TAMs-related markers including CD8, CD4, Fox3P, and CD25 were used to analyze T cells and Tregs. The tumor tissue was finely minced and cells were isolated after centrifugation. Continuously, the cells were blocked with 1 mL of 5% FBS and incubated for 30 min. The supernatant was removed after centrifugation, 35 μL of 5% FBS and 0.2 μL of fluorescent antibodies were added to the cells and incubated for 30 min at room temperature while avoiding lights. Then, the stained cells were centrifuged at 600 g for 5 min and the supernatant was removed. Using 300 μL of 1X PBS, the cells were washed three times and measured fluorescence intensity by FACS.

**Genes**

c-MYC (NP_001170823.1) and BCL2 (NP_803129.2) genes were searched by PubMed (<https://www.ncbi.nlm.nih.gov/gene/>) and contrasted with miRNAs from GENs.

**Lipid nanoparticle formulation and transfection *in vitro***

To utilize GENs as a nanovector, lipids were extracted from GENs. 1 μL of GENs lipid, 3 μL of Opti-MEM™ and 10 nM of miRNAs were mixed. Then, the solution was treated under a bath sonication for 20 min. 5 μL of GENs and miRNAs were added in 1 × 10^4^ C6 glioma cells and incubated for 48 h at 37 ℃.

0.2 μL of 10 μM of Lipo 8000 (China, Shanghai, Beyotime), 3 μL of Opti-MEM™ and 10 nM of miRNAs were gently mixed and incubated at room temperature for 20 min. Then, the mixture was applied to cells.

**Lipid extraction from GENs**

The lipid extraction method followed by the Bligh and Dyer lipid extraction protocol. [4] Briefly described, a mixture of chloroform and methanol (2:1, v/v) was prepared and 0.1g of GENs with 1 mL of water was added into the solution. (Final volume; 86:14:1; v/v/v: chloroform/methanol/water) After vortexing, the solution of the lower phase containing lipids was achieved. Then, it was treated under a dry vacuum at 48℃ for 10 min.

**Encapsulation efficiency**

The encapsulation efﬁciency was calculated as the percentage of miRNAs encapsulated in GENs versus the total amount of miRNAs used initially. The drug-loading amount was presented as the percentage of the weight of miRNAs encapsulated in the GENs versus the total weight of GENs used initially. The encapsulation efﬁciency and drug-loading rates were represented by the following equations:

Encapsulation efﬁciency (EE) = $\frac{miRNAs encapsulated in GENs}{miRNAs in GENs}$ x 100%

Drug-loading amount (DL) = $\frac{The weight of miRNAs encapsulated in GENs}{The total weight of GENs}$ x 100%

**GO and KEGG enrichment analysis**

Gene Ontology (GO) enrichment analysis was used on the target gene candidates of differentially expressed miRNAs (‘target gene candidates’ in the following). GO seq based Wallenius non-central hyper-geometric distribution [5]，which could adjust for gene length bias, was implemented for GO enrichment analysis. KEGG [6] is a database resource for understanding high-level functions and utilities of the biological system, such as the cell, the organism and the ecosystem, from molecular-level information, especially large-scale molecular datasets generated by genome sequencing and other high-throughput experimental technologies (http://www.genome.jp/kegg/). We used KOBAS [7] software to test the statistical enrichment of the target gene candidates in KEGG pathways.

**Reference**

1. Kluza E, Yeo SY, Schmid S, van der Schaft DW, Boekhoven RW, Schiffelers RM, Storm G, Strijkers GJ, Nicolay K: Anti-tumor activity of liposomal glucocorticoids: The relevance of liposome-mediated drug delivery, intratumoral localization and systemic activity**.** J Control Release 2011;151(1)**:**10-17.

2. Floris A, Sinico C, Fadda AM, Lai F, Marongiu F, Scano A, Pilloni M, Angius F, Vazquez-Vazquez C, Ennas G: Characterization and cytotoxicity studies on liposome-hydrophobic magnetite hybrid colloids**.** J Colloid Interface Sci 2014;425118-127.

3. Zhu Y, Liang J, Gao C, Wang A, Xia J, Hong C, Zhong Z, Zuo Z, Kim J, Ren H, et al: Multifunctional ginsenoside Rg3-based liposomes for glioma targeting therapy**.** J Control Release 2021;330641-657.

4. Breil C, Abert Vian M, Zemb T, Kunz W, Chemat F: "Bligh and Dyer" and Folch Methods for Solid-Liquid-Liquid Extraction of Lipids from Microorganisms. Comprehension of Solvatation Mechanisms and towards Substitution with Alternative Solvents**.** Int J Mol Sci 2017;18(4).

5. Young MD, Wakefield MJ, Smyth GK, Oshlack A: Gene ontology analysis for RNA-seq: accounting for selection bias**.** Genome Biol 2010;11(2)**:**R14.

6. Kanehisa M, Araki M, Goto S, Hattori M, Hirakawa M, Itoh M, Katayama T, Kawashima S, Okuda S, Tokimatsu T, Yamanishi Y: KEGG for linking genomes to life and the environment**.** Nucleic Acids Res 2008;36(Database issue)**:**D480-484.

7. Mao X, Cai T, Olyarchuk JG, Wei L: Automated genome annotation and pathway identification using the KEGG Orthology (KO) as a controlled vocabulary**.** Bioinformatics 2005;21(19)**:**3787-3793.


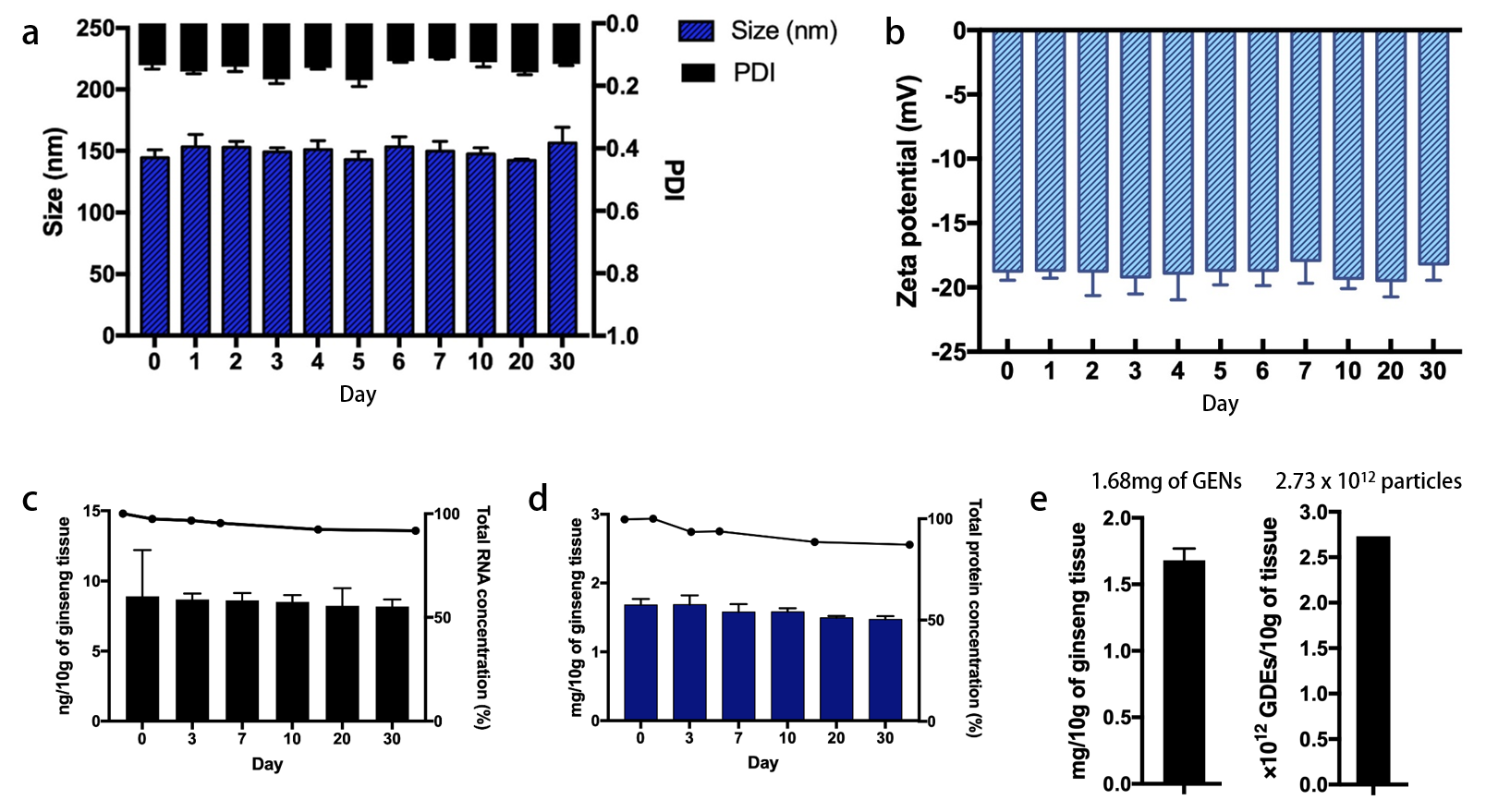


**Additional file 1: Fig. S1. Stability and characterization of GENs.** (a)(b) Size, PDI, and zeta potential measurement of GENs for 30 days. (c) Total RNA concentration of GENs. (d) Total protein concentration of GENs. (e) Quantification of the production of GENs based on the protein concentration. 1.68mg of GENs can be achieved by 10g of ginseng. (Left) Using nano tracking analysis (NTA), the nanoparticles of GENs were counted. (Right)


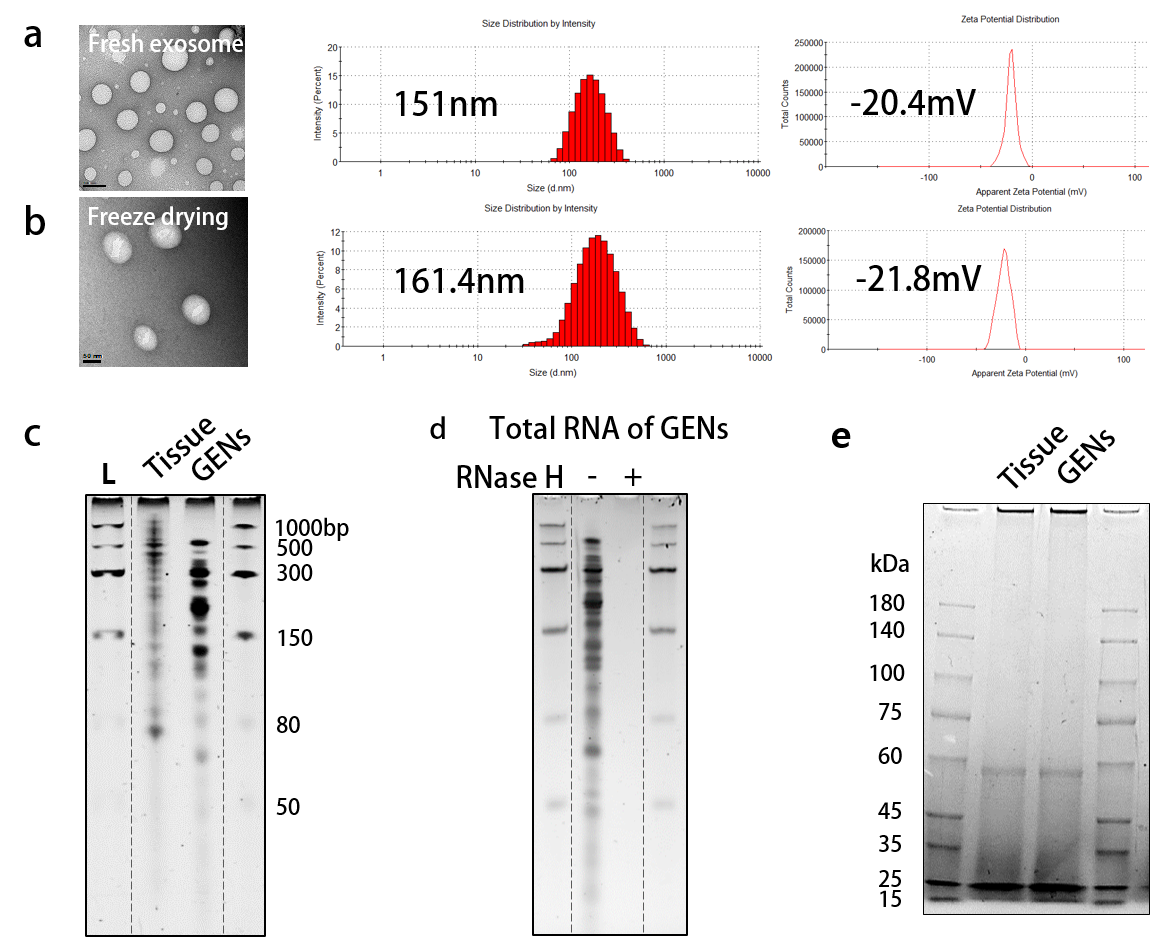


**Additional file 1: Fig. S2. Characterization of GENs.** (a)(b) Before and after the freeze-drying of GENs, the size and zeta potential were measured. (c) Total RNA was extracted from ginseng tissue and GENs. (d) Total RNA extracted from GENs was treated with RNase H and observed by 10% PAGE 8M urea gel. (e) Total proteins of ginseng tissue and GENs.


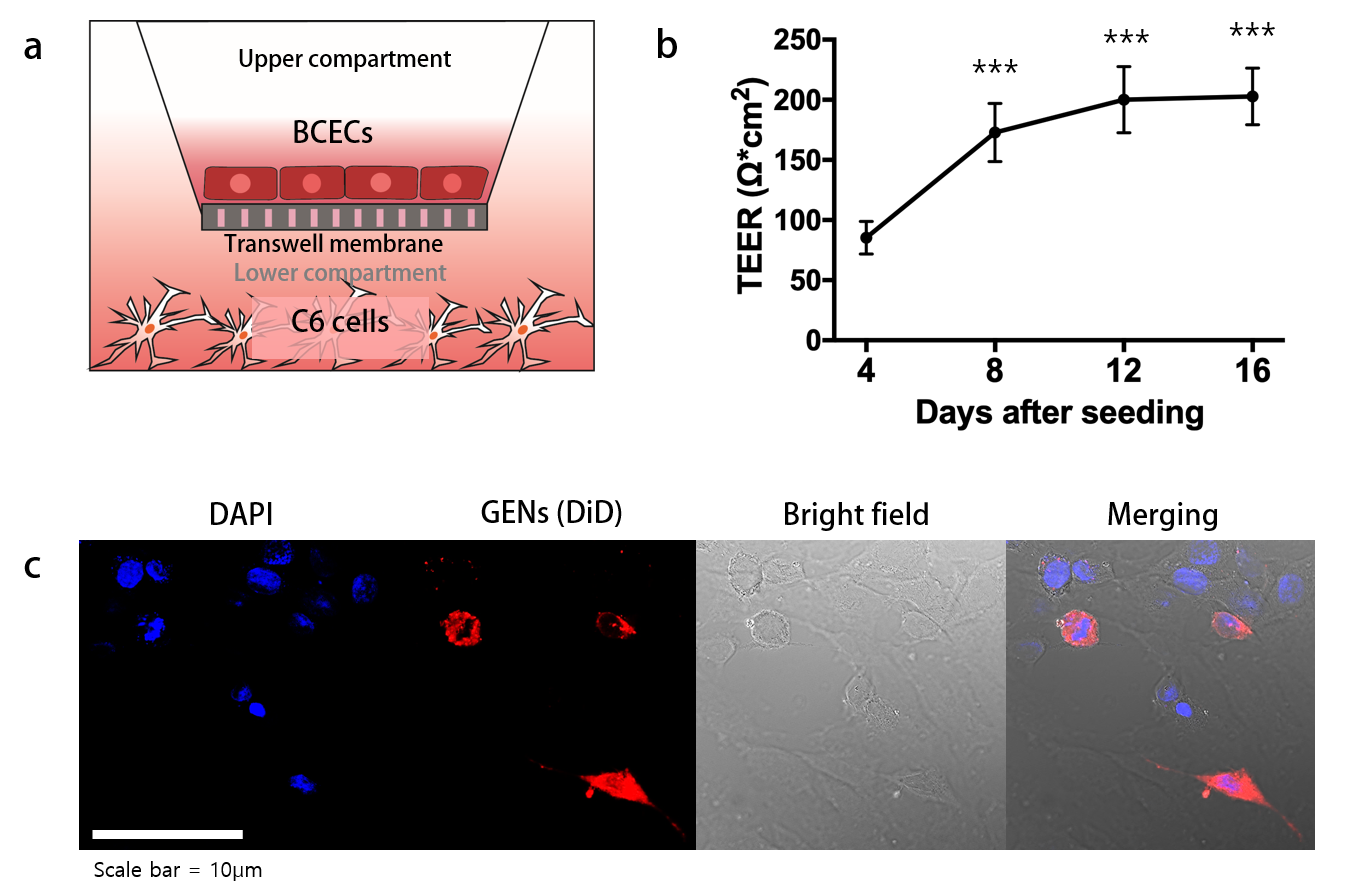


**Additional file 1: Fig. S3. Transwell of C6 glioma cells and BCECs.** (a) A schematic illustration of the fabrication of transwell *in vitro*. BCECs were seeded on 0.4 µm of transwell, and C6 glioma cells were seeded on the lower compartment of the transwell (b) TEER measurement to confirm the integrity and permeability of epithelial cells for 16 days. (c) Cellular uptake of DiD-labeled GENs by C6 glioma cells through transmembrane with 12 hr of incubation. (***: P<0.001)


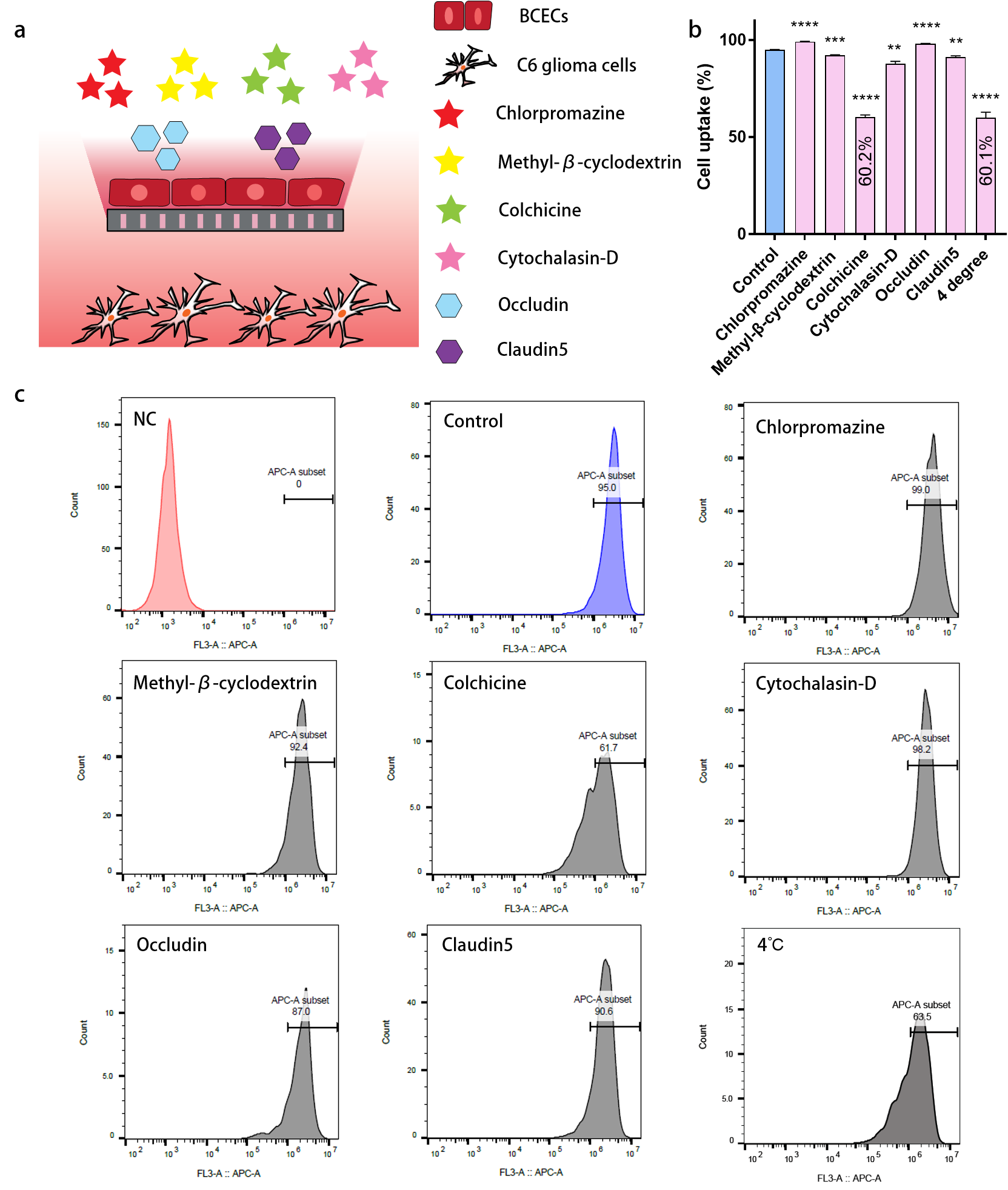


**Additional file 1: Fig. S4. Blocking BBB endocytosis of DiD-labeled GENs in C6 glioma cells *in vitro.*** (a)(b) BBB transmembrane model with inhibitors including chlorpromazine, methyl-β-cyclodextrin, colchicine, cytochalasin-D, occludin, and claudin-5. (c) Flow cytometry analysis of cellular uptake in C6 glioma cells by DiD-labeled GENs through BBB membrane seeded BCECs. The experiment was undertaken in triplicate. (**: P<0.01, ***: P<0.001, ****: P<0.0001)


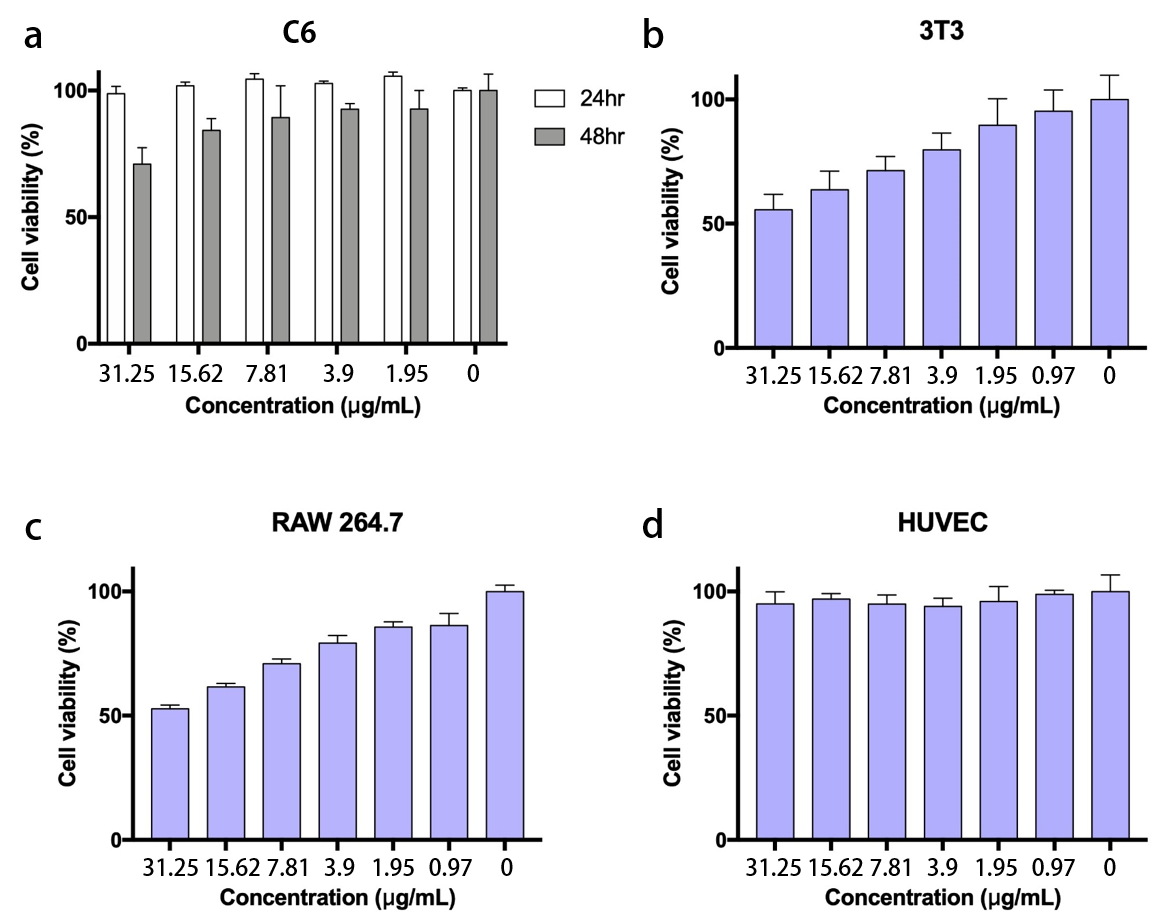


**Additional file 1: Fig. S5. Cell viability of GENs was individually evaluated by MTT assay in C6 glioma, 3T3, RAW 264.7, and HUVEC. (n = 6)**

**
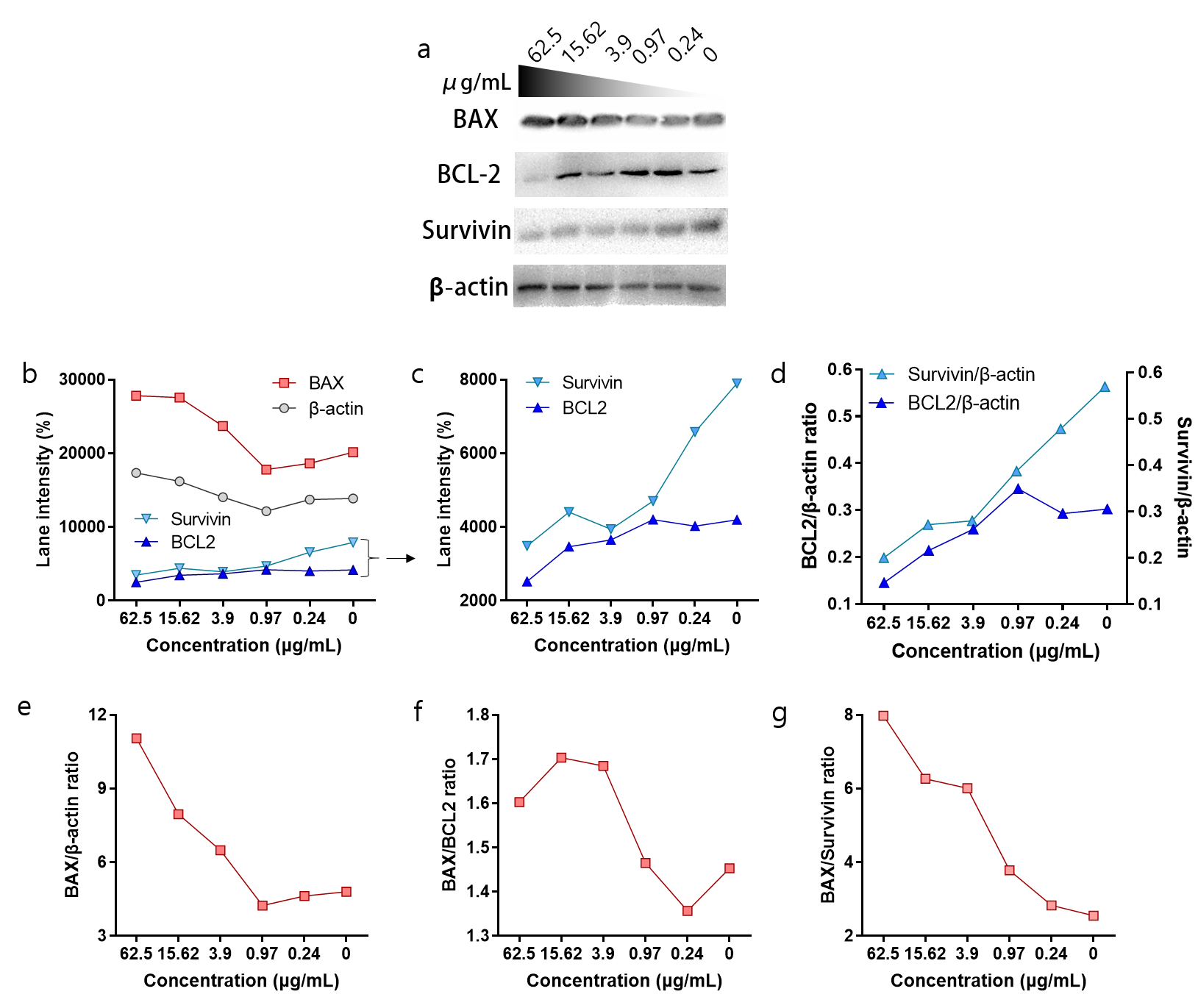
 Additional file 1: Fig. S6. Western blotting of protein expression of apoptosis-related proteins with sequential reduction of GENs’ concentrations from 62.5 to 0.24 μg/mL.** (a) The number of proteins loaded into 12% SDS-PAGE gels. Western blotting of BAX, BCL-2, Survivin, and β-actin. (b) Lane intensity (%) of apoptosis-related proteins. (c) Lane intensity of Survivin and BCL-2 in the narrow intensity range. (d) The relative abundance ratio of immunoreactivity of BCL-2 and Survivin. (e)-(g) Relative abundance ratio of immunoreactivity of BAX represented by dividing β-actin, BCL-2, and Survivin.


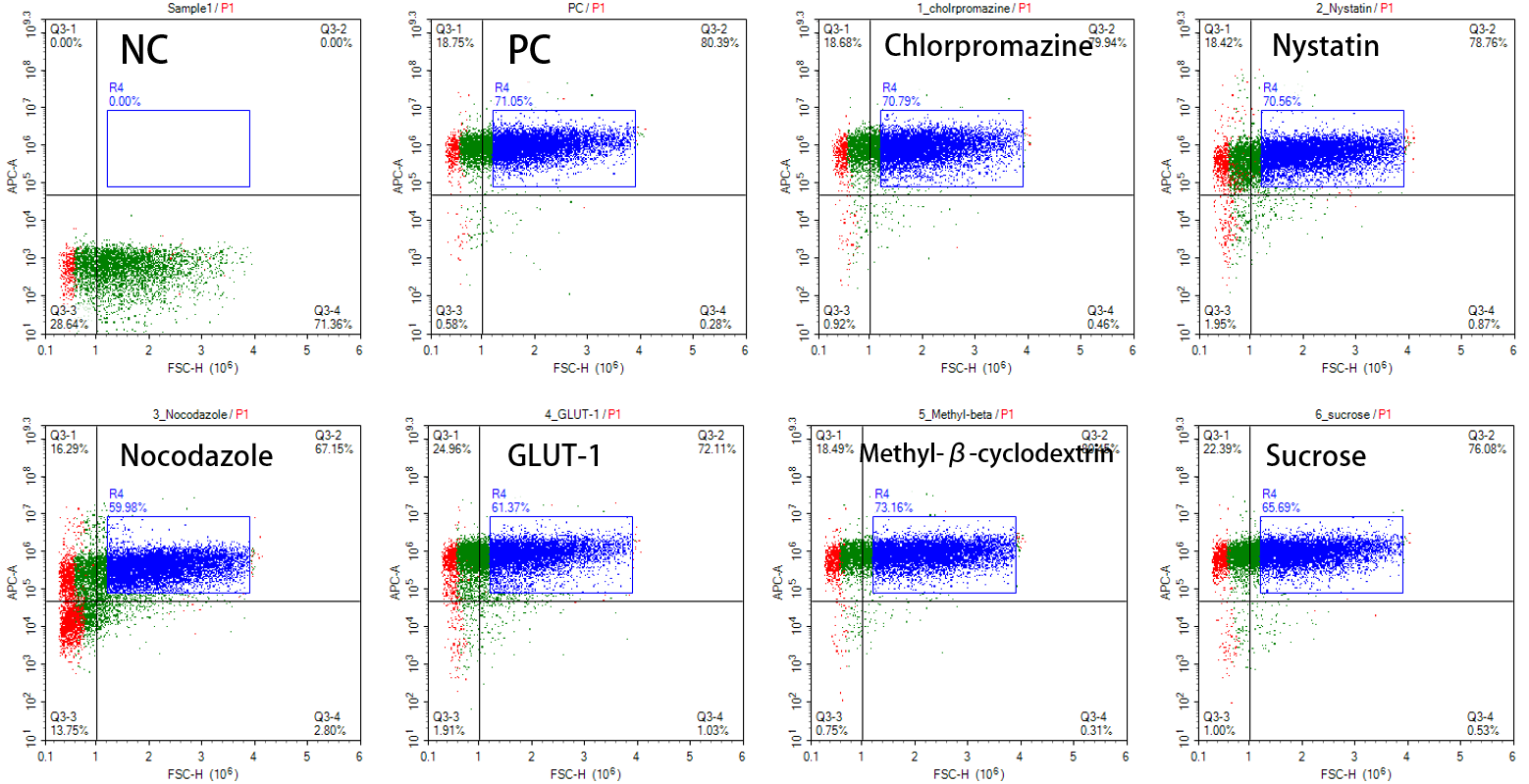


**Additional file 1: Fig. S7. Inhibition of endocytosis of GENs with chemical inhibitors in C6 glioma cell**. C6 glioma cell was inhibited by using inhibitors, and the cellular uptake of DiD-labeled GENs was measured by flow cytometry.


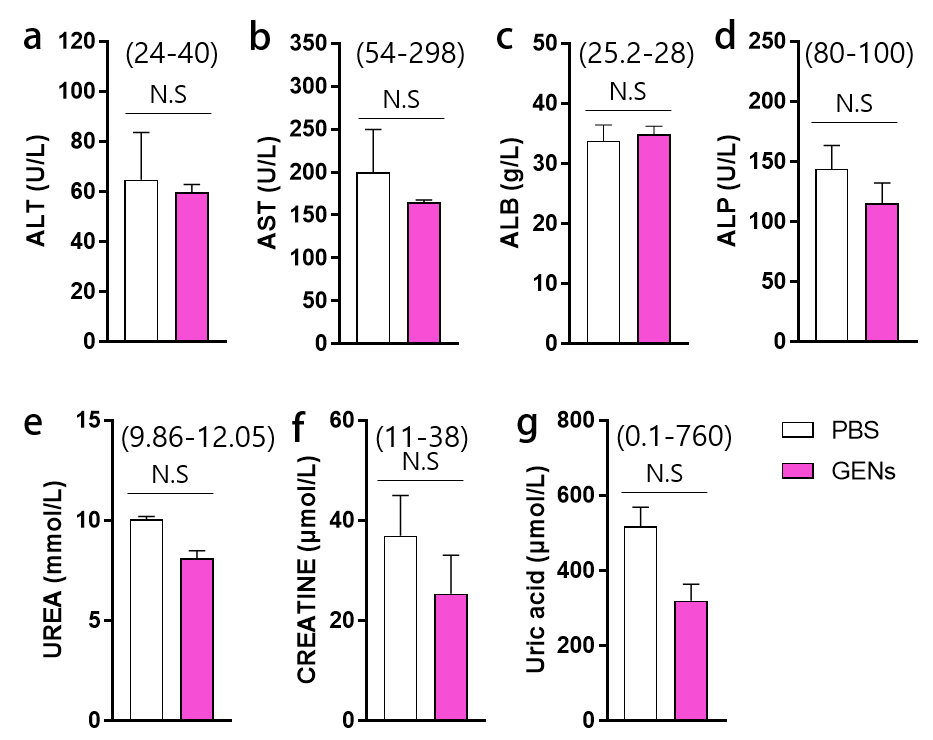


**Additional file 1: Fig. S8. Blood chemistry analysis in serum *in vivo*.** (a-d) Evaluation of liver toxicity. (e-g) Evaluation of kidney function by blood urea nitrogen, serum creatinine, and uric acid. Data are expressed as the mean±S.D. (N=3, per group)


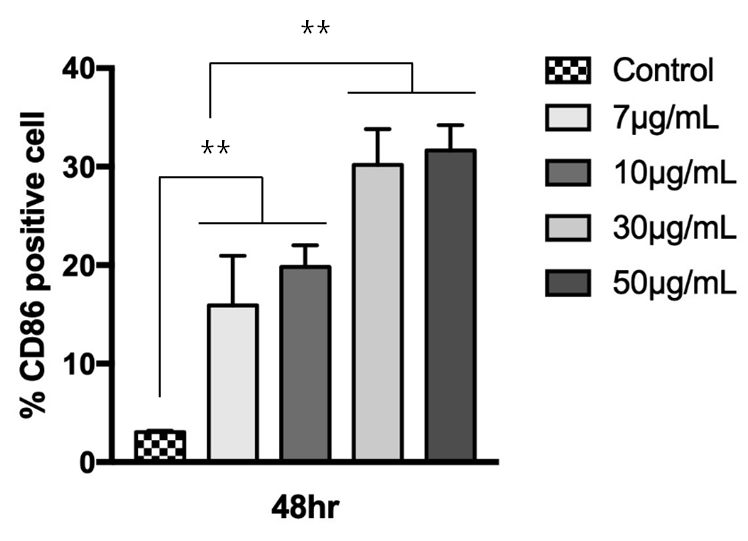


**Additional file 1: Fig. S9. Flow cytometry analysis of CD86 expression in RAW 264.7 with the treatment of different concentrations of GENs.** RAW 264.7 was stimulated by 100 ng/mL of LPS and 50 ng/mL of INF-γ for 48 h and consistently treated by GENs for 48 h. (**: P<0.01)

**
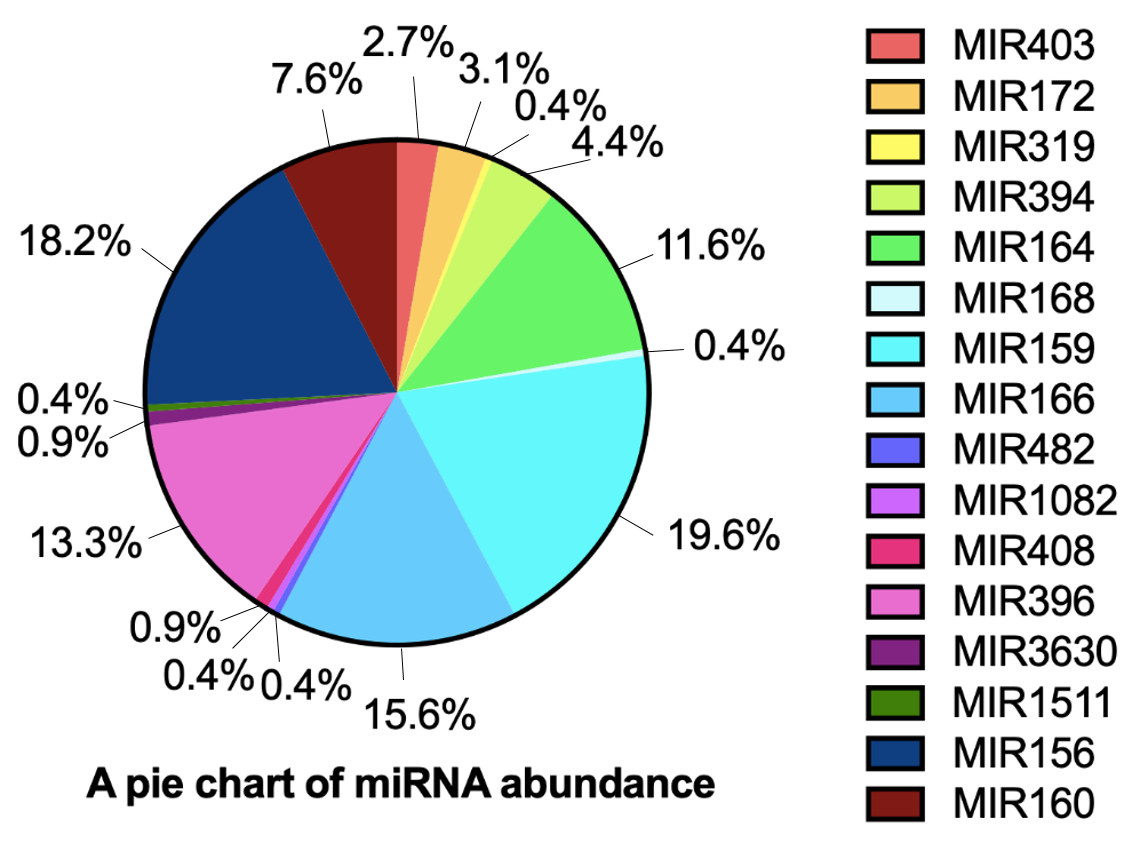
**

**Fig. S10. A pie chart of miRNA abundance in GENs.** Distribution of the miRNA abundance by miRNA family. (MIR403; aly-MIR403a, ath-MIR403, bra-MIR403, gma-MIR403a, ptc-MIR403c, sly-MIR403. MIR172; ath-MIR172c, ath-MIR172d, bra-MIR172c, gra-MIR172b, lus-MIR172j, mtr-MIR172d, ptc-MIR172h. MIR319; mtr-MIR319c. MIR394; aly-MIR394a, aly-MIR394b, ath-MIR394a, ath-MIR394b, gma-MIR394a, gma-MIR394b, ptc-MIR394a, sly-MIR394, stu-MIR384, zma-MIR394a. MIR164; aly-MIR164a, aly-MIR164c, ata-MIR164a, ata-MIR164b, ata-MIR164c, ath-MIR164a, ath-MIR164b, ath-MIR164c, bdi-MIR164a, bdi-MIR164c, bdi-MIR164f, bra-MIR164e, gma-MIR164b, osa-MIR164d, osa-MIR164e, rgl-MIR164, sly-MIR164a, sly-MIR164b vun-MIR164, zma-MIR164a, zma-MIR164b, zma-MIR164c, zma-MIR164d, zma-MIR164f, zma-MIR164g, zma-MIR164h. MIR168; sly-MIR168a. MIR159; aly-MIR159a, aly-MIR159c, aly-MIR319a, aly-MIR319b, aly-MIR319c, aqc-MIR159, ath-MIR159a, ath-MIR159b, ath-MIR159c, ath-MIR319a, ath-MIR319c, bdi-MIR159a, bdi-MIR159b, bdi-MIR319a, gma-MIR159a, gma-MIR159e, gma-MIR319c, gma-MIR319q, lus-MIR159b, mdm-MIR159a, mdm-MIR159c, mdm-MIR319b, mes-MIR159a, mtr-MIR319a, mtr-MIR319b, osa-MIR159a, osa-MIR159c, osa-MIR159d, osa-MIR159e, osa-MIR159f, ppt-MIR319a, ppt-MIR319d, pta-MIR159a, pta-MIR159c, pta-MIR319, pvu-MIR159a, sly-MIR319c, smo-MIR319, sof-MIR159c, stu-MIR319a, zma-MIR159a, zma-MIR159b, zma-MIR159c, zma-MIR159f. MIR166; aly-MIR166f, aly-MIR166g, ata-MIR166b, ata-MIR166c, ath-MIR166a, ath-MIR166e, bdi-MIR166a, bdi-MIR166i, cme-MIR166i, cpa-MIR166e, csi-MIR166c, csi-MIR166d, gma-MIR166i, gma-MIR166m, gma-MIR166u, gra-MIR166c, gra-MIR166d, mtr-MIR166b, osa-MIR166a, osa-MIR166b, osa-MIR166c, osa-MIR166d, osa-MIR166g, osa-MIR166h, osa-MIR166i, osa-MIR166j, osa-MIR166m, ppt-MIR166j, ptc-MIR166n, sly-MIR166c, stu-MIR166d, vvi-MIR166a, zma-MIR166h, zma-MIR166i, zma-MIR166l. MIR482; pgi-MIR482a, MIR1082; smo-MIR1082a, MIR408; aly-MIR408, osa-MIR408. MIR396; ama-MIR396, ath-MIR396a, ath-MIR396b, atr-MIR396d, cca-MIR396a, cca-MIR396c, csi-MIR396a, csi-MIR396c, gma-MIR396a, gma-MIR396b, gma-MIR396d, gma-MIR396f, gma-MIR396g, gma-MIR396h, mdm-MIR396a, mtr-MIR396c, osa-MIR396a, osa-MIR396c, pab-MIR396a, pde-MIR396, ppe-MIR396a, ptc-MIR396e, ptc-MIR396f, ptc-MIR396g, smo-MIR396, stu-MIR396, vvi-MIR396a, vvi-MIR396b, zma-MIR396e, zma-MIR396g. MIR3630; han-MIR3630, vvi-MIR3630. MIR1511; ppe-MIR1511. MIR156; ahy-MIR156b, aly-MIR156b, aly-MIR156d, aly-MIR156e, aly-MIR156f, aly-MIR157b, ata-MIR156b, ata-MIR156d, ath-MIR156a, ath-MIR156b, ath-MIR156c, ath-MIR156d, ath-MIR156f, ath-MIR157a, ath-MIR157c, bdi-MIR156b, bdi-MIR156d, bdi-MIR156f, bdi-MIR156g, bdi-MIR156h, cca-MIR156b, gma-MIR156g, hbr-MIR156, mtr-MIR156b, mtr-MIR156c, mtr-MIR156d, mtr-MIR156h, mtr-MIR156i, osa-MIR156b, osa-MIR156c, osa-MIR156f, osa-MIR156j, osa-MIR156l, stu-MIR156f, stu-MIR156g, zma-MIR156a, zma-MIR156b, zma-MIR156e, zma-MIR156h, zma-MIR156i, zma-MIR156l. MIR160; aau-MIR160, aly-MIR160b, aly-MIR160c, ata-MIR160a, ata-MIR160b, ath-MIR160a, ath-MIR160c, ghr-MIR160, gma-MIR160a, gma-MIR160b, osa-MIR160a, osa-MIR160c, osa-MIR160d, osa-MIR160e, zma-MIR160c, zma-MIR160d, zma-MIR160f.)


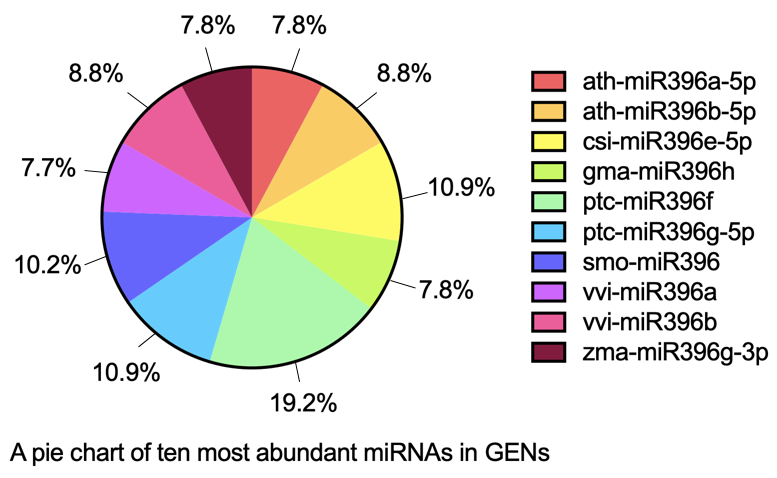


**Additional file 1: Fig. S11. A pie chart of the ten most abundant miRNAs in GENs.**


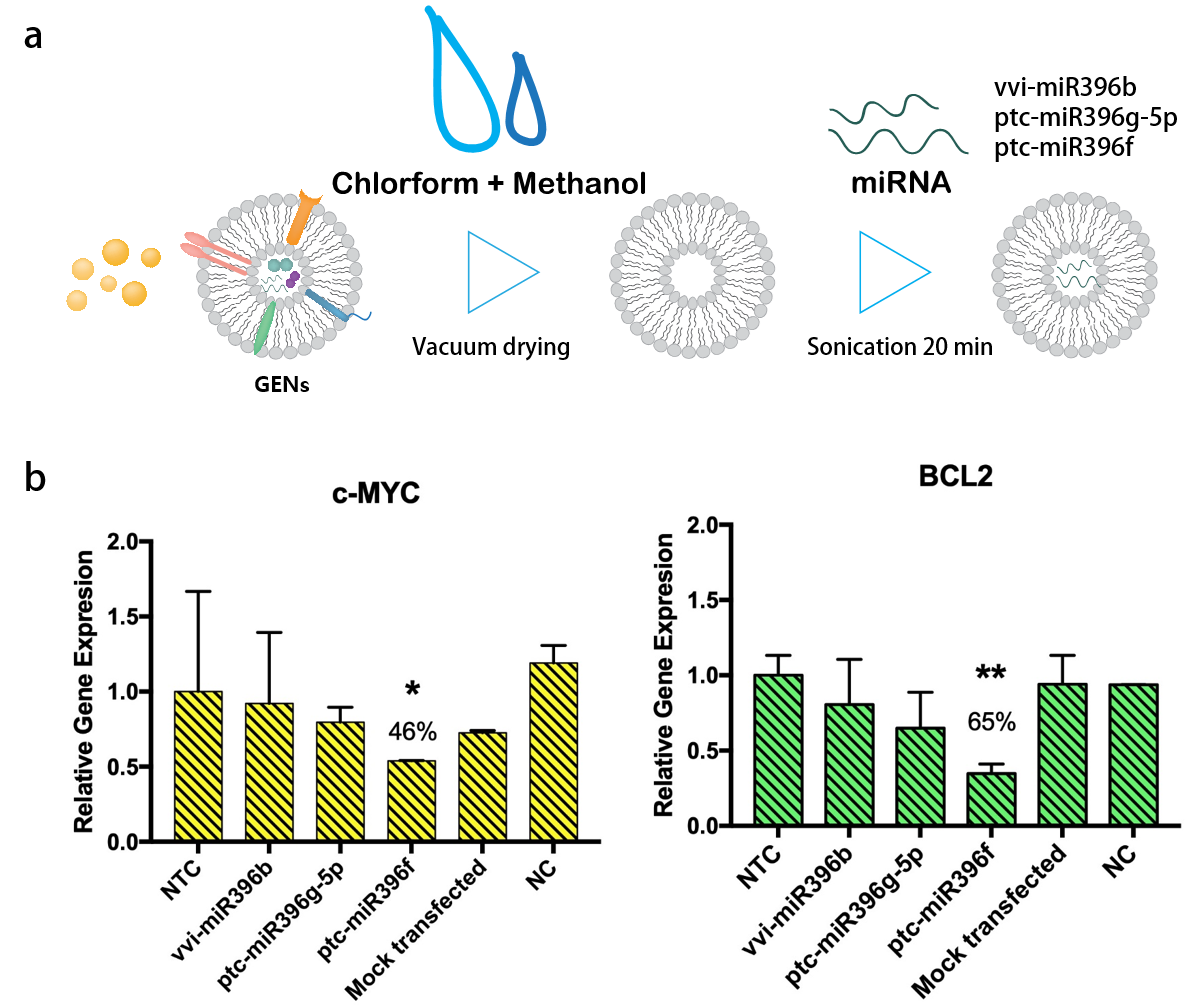


**Fig. S12. Lipid extraction from GENs and the exosomal delivery of miRNAs.** (a) Extraction of exosomal lipid from GENs by Bligh and Dyer lipid extraction protocol and transfection of the lipid. (b) Transfection of GENs lipid and miRNAs in C6 glioma cell. (*: P<0.05, **: P<0.01)

|  | GENs |
| --- | --- |
| Encapsulation efﬁciency (EE, %) | 91.3 ± 17.9 |

**Fig. S13. Encapsulation of miRNA in GENs.** The encapsulation of miRNA into GENs by sonication.

**
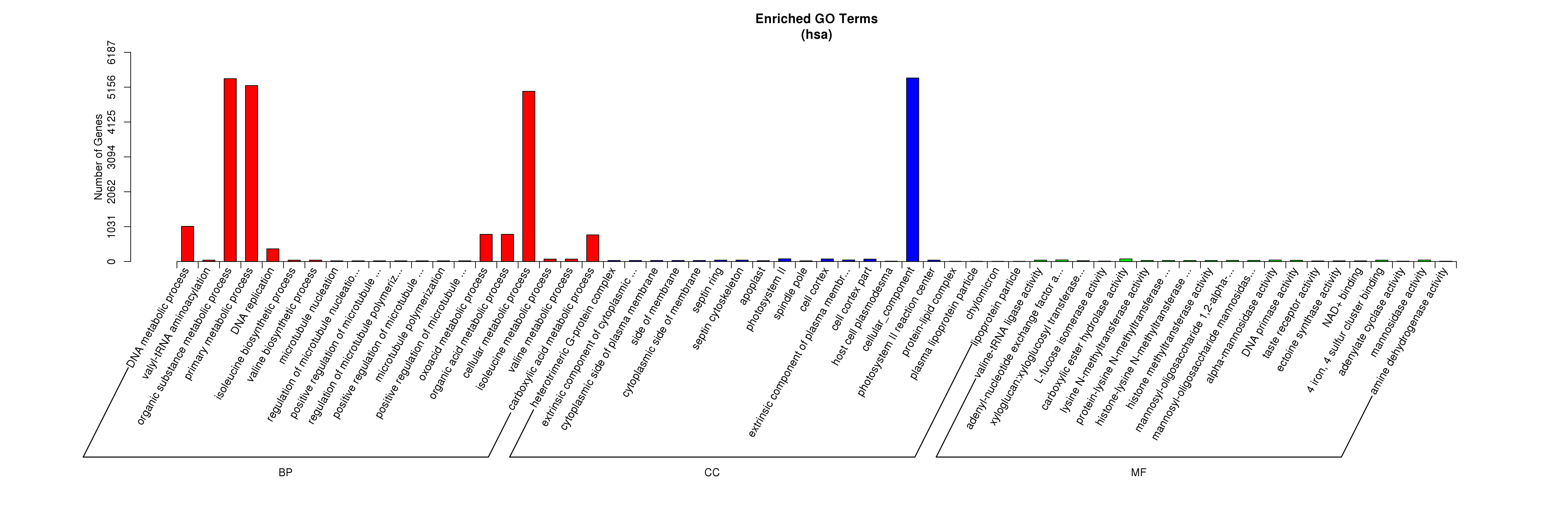
**

**Fig. S14. Enriched gene ontology (GO) terms (hsa).** Identification of overrepresented GO terms. The X-axis indicates the GO categories, and the Y-axis indicates the number of differentially expressed genes in a GO category. Color coding is used to identify the molecular function, cellular component, and biological process categories. (BP: biological process, MF: molecular function, and CC: cellular component.)

**
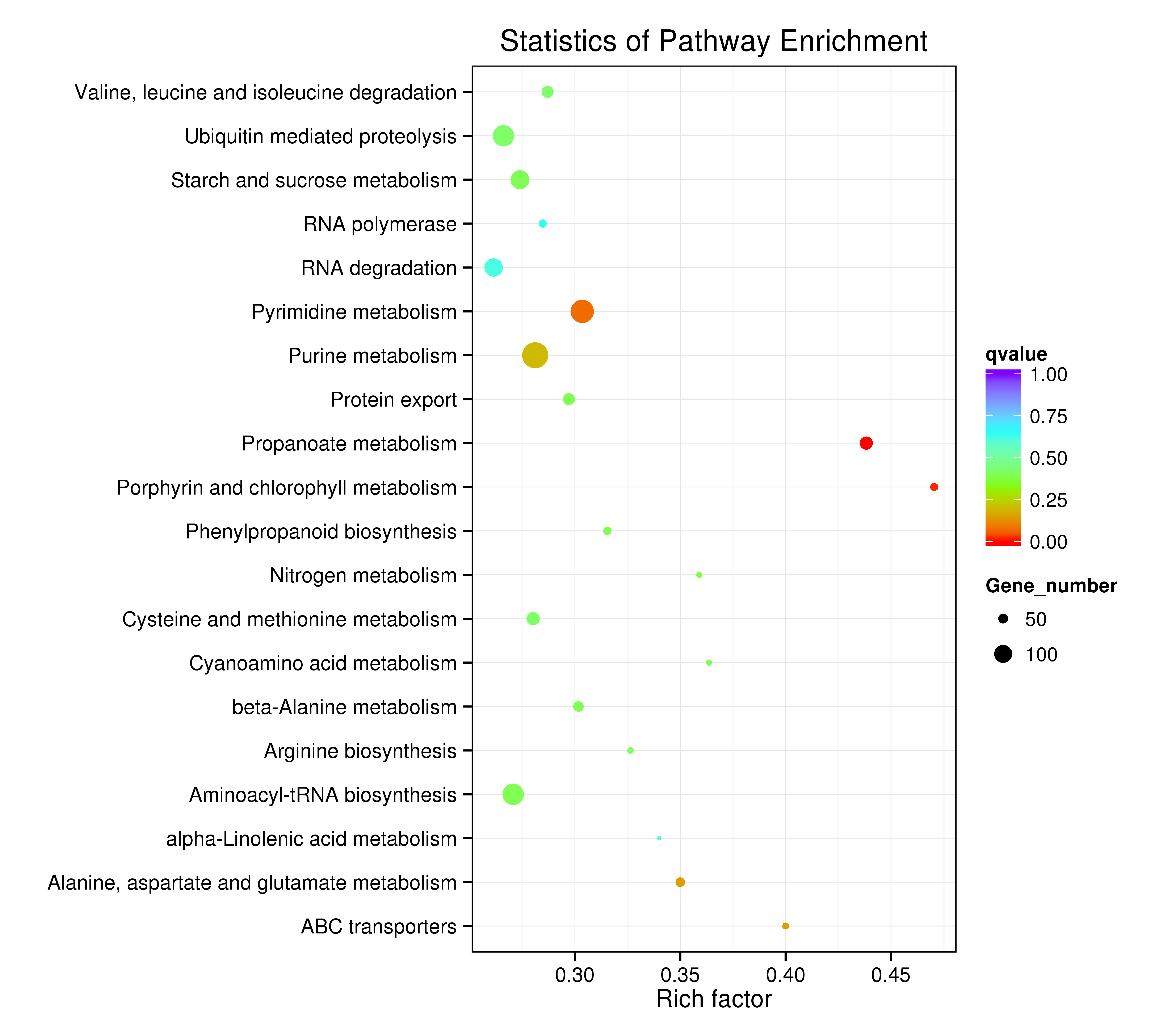
**

**Fig. S15. Gene Kyoto Encyclopedia of Genes and Genomes (KEGG) enrichment analysis of GENs.** The X-axis represents the enriched factor and the ratio of the number of differentially expressed genes in the pathway to the total number of genes in this pathway, and the Y-Axis represents the KEGG pathway. The color of the dot represents the qvalue. The size represents the number of differentially expressed genes contained in each pathway. The qvalue is the P-value after multiple hypothesis test corrections and in the range between 0 and 1. The closer the qvalue is to zero, the more significant the enrichment is. The mapping resulted in significantly enriched terms in 20 pathways.

**Additional file 1: Table S1. Primer set list**

|  | Forward (5’->3’) | Reverse (5’->3’) |
| --- | --- | --- |

| Bax | AGG GTT TCA TCC AGG ATC GAG C | AGG CGG TGA GGA CTC CAG CC |
| --- | --- | --- |
| Bcl-2 | TCG CTC TGT GGA TGA CTG AG | CAG CGT CTT CAG AGA CAG CCA |
| Bcl-xL | GAA TCT TAT CTT GGC TTT GGA | GTA GAG TGG ATG GTC AGT GT |
| Survivin | TTC TCA AGG ACC ACC GCA TC | GCCAAGTCTGGCTCGTTCTC |
| GAPDH | CTC ATG ACC ACA GTC CAT GC | TAC ATT GGG GGT AGG AAC AC |
| c-MYC | GAA AAC GAC AAG AGG CGG AC | AAT GGA CAG GAT GTA GGC GG |
| iNOS | CAG AGG ACC CAG AGA CAA GC | TGC TGA AAC ATT TCC TGT GC |
| IL-6 | CCC CAA TTT CCA ATG CTC TCC | TTG GTC CTT AGC CAC TCC TTC |
| CCL 2 | CTT CTG GGC CTG CTG TTC A | CCA GCC TAC TCA TTG GGA TCA |
| CCL 3 | CAT ATG GAG CTG ACA CCC CG | GAG CAA AGG CTG CTG GTT TC |
| CD 86 | TCA ATG GGA CTG CAT ATC TGC C | GCC AAA ATA CTA CCA GCT CAC T |
| CD206 | CAG GTG TGG GCT CAG GTA GT | TGT GGT GAG CTG AAA GGT GA |
| IL-10 | CTT ACT GAC TGG CAT GAG GAT CA | GCA GCT CTA GGA GCA TGT GG |
| F4/80 | TAT CTT TTC CTC GCC TGC TTC | CAC CAC CTT CAG GTT TCT CAC |
| TGFB1 | TGG CTG AAC CAA GGA GAC GG | TTG GGG CTG ATC CCG TTG AT |
| TGFB2 | AAG TCC TCA GCC TGT ACA ACA C | GCT GTT CGA TCT TGG GCG TA |
| TGFB3 | ATG ACC CAC GTC CCC TAT CA | ACT CAG ACT CCG AGG TCT CC |
| EGF | GAATTCAGTTGCCCTGACTCTACCGC | GAATTCGCAGACAGCCACCATGAT |
| CXCL12 | TGG CTC TGC TCG CCT TTG C | GGA CCC CAG CAC AGA TAC TT |
| CXCL14 | GCT TAG CCA GTG CAG AAG GA | CAC TGT GTT CTG GCG TTT GG |
| VEGF | GGA GAT CCT TCG AGG AGC ACT T | GGC GAT TTA GCA GCA GAT ATA AGA A |
| STAT3 | GAA AAC GAC AAG AGG CGG AC | AAT GGA CAG GAT GTA GGC GG |
| ITGAV | GGGATGACAACCCTCTGAC | GTCACATACCACCTGGCGA |
| ITGB6 | GCAGAACGCTCTAAGGCCAA | AGCGGAGTTGACTTTCTCTG |
| ITGB8 | GGATGGTGTGTTCAAGAGGA | TCAGCTCCTGGATGCAGTTG |
| ITGB1 | GAGGTTCAATTTGAAATTAGC | AAATGTCCCATTTCCCTCATG |
| ITGB3 | GGACATCTACTACTTGATGG | ATACAGGCTTGTCCACGAAG |

**Additional file 1: Table S2. RNA Profiling Analysis**

|  | **Mature** |  | **Mature** |  | **Mature** |  | **Mature** |
| --- | --- | --- | --- | --- | --- | --- | --- |
| 1 | aau-miR160 | 26 | cca-miR396a-3p | 51 | gma-miR6300 | 76 | ppe-miR396a |
| 2 | aof-miR5139b | 27 | cca-miR396c | 52 | han-miR3630-3p | 77 | ppe-miR1511-3p |
| 3 | Aqc-miR159 | 28 | cme-miR166i | 53 | hbr-miR156 | 78 | ppt-miR166j |
| 4 | ath-miR156a-5p | 29 | cpa-miR166e | 54 | hci-miR164a | 79 | ppt-miR319a |
| 5 | ath-miR157a-5p | 30 | cpa-miR8155 | 55 | lja-miR166-3p | 80 | pta-miR159a |
| 6 | ath-miR159a | 31 | csi-miR156d-5p | 56 | lus-miR159b | 81 | pta-miR159c |
| 7 | ath-miR159b-3p | 32 | csi-miR160a-5p | 57 | mdm-miR159a | 82 | pta-miR319 |
| 8 | ath-miR159b-3p | 33 | csi-miR160c-5p | 58 | mdm-miR396a | 83 | ptc-miR166n |
| 9 | ath-miR159c | 34 | csi-miR396a-3p | 59 | mtr-miR166b | 84 | ptc-miR396f |
| 10 | ath-miR160a-5p | 35 | csi-miR396e-5p | 60 | mtr-miR319a-3p | 85 | ptc-miR396g-5p |
| 11 | ath-miR164a | 36 | csi-miR403b-5p | 61 | osa-miR159a.1 | 86 | rgl-miR5139 |
| 12 | ath-miR164c-5p | 37 | fve-miR156h | 62 | osa-miR159c | 87 | sly-miR403-5p |
| 13 | ath-miR166a-3p | 38 | fve-miR159b | 63 | osa-miR159d | 88 | smo-miR396 |
| 14 | ath-miR172c | 39 | fve-miR159c | 64 | osa-miR159e | 89 | sof-miR159c |
| 15 | ath-miR319a | 40 | gma-miR160b | 65 | osa-miR159f | 90 | stu-miR156f-5p |
| 16 | ath-miR319c | 41 | gma-miR164b | 66 | osa-miR160e-5p | 91 | vvi-miR166a |
| 17 | ath-miR394a | 42 | gma-miR166m | 67 | osa-miR164d | 92 | vvi-miR396a |
| 18 | ath-miR396a-3p | 43 | gma-miR166u | 68 | osa-miR164e | 93 | vvi-miR396b |
| 19 | ath-miR396a-5p | 44 | gma-miR319c | 69 | osa-miR166g-3p | 94 | vvi-miR3630-3p |
| 20 | ath-miR396b-5p | 45 | gma-miR319q | 70 | osa-miR166i-3p | 95 | zma-miR164h-5p |
| 21 | ath-miR403-3p | 46 | gma-miR396a-3p | 71 | osa-miR166m | 96 | zma-miR166h-3p |
| 22 | atr-miR396d | 47 | gma-miR396h | 72 | osa-miR396a-3p | 97 | zma-miR396g-3p |
| 23 | bdi-miR159a-3p | 48 | gma-miR403a | 73 | pde-miR396 | 98 | zma-miR396g-5p |
| 24 | bdi-miR845 | 49 | gma-miR4995 | 74 | peu-miR2916 |  |  |
| 25 | cca-miR156b | 50 | gma-miR5368 | 75 | pgi-miR482a |  |  |

**Additional file 1: Table S3. Protein profiles of GENs**

| **Accession** | **Description** | **Score** | **Coverage** | **# Proteins** | **# Unique Peptides** | **# Peptides** | **# PSMs** | **# AAs** | **MW [kDa]** | **calc. pI** |
| --- | --- | --- | --- | --- | --- | --- | --- | --- | --- | --- |
| P83618 | Ribonuclease-like storage protein OS=Panax ginseng OX=4054 PE=1 SV=2 - [RN28_PANGI] | 2130.18 | 46.64 | 1 | 11 | 11 | 59 | 238 | 27.3 | 6.29 |
| D0VFU1 | Glyceraldehyde-3-phosphate dehydrogenase (Fragment) OS=Panax ginseng OX=4054 PE=4 SV=1 - [D0VFU1_PANGI] | 1427.85 | 64.06 | 2 | 2 | 11 | 28 | 217 | 23.3 | 5.96 |
| A0A060ILK2 | Glyceraldehyde-3-phosphate dehydrogenase (Fragment) OS=Panax ginseng OX=4054 GN=GAPDH PE=2 SV=1 - [A0A060ILK2_PANGI] | 1417.80 | 47.99 | 1 | 4 | 12 | 29 | 298 | 32.2 | 7.12 |
| A0A096VPM7 | Pleiotropic drug resistance transporter 1 OS=Panax ginseng OX=4054 GN=PDR1 PE=2 SV=1 - [A0A096VPM7_PANGI] | 1116.32 | 17.48 | 2 | 14 | 23 | 33 | 1447 | 162.4 | 6.74 |
| Q6VAL5 | Glyceraldehyde-3-phosphate dehydrogenase OS=Panax ginseng OX=4054 PE=2 SV=1 - [Q6VAL5_PANGI] | 1026.99 | 46.10 | 1 | 4 | 11 | 22 | 295 | 31.8 | 6.89 |
| A0A060IMN8 | Actin 1 OS=Panax ginseng OX=4054 GN=ACT1 PE=2 SV=1 - [A0A060IMN8_PANGI] | 819.71 | 52.52 | 3 | 5 | 14 | 23 | 377 | 41.7 | 5.49 |
| D3JX88 | Glutamate decarboxylase OS=Panax ginseng OX=4054 GN=GAD PE=2 SV=1 - [D3JX88_PANGI] | 630.45 | 28.43 | 1 | 11 | 11 | 14 | 496 | 56.0 | 5.96 |
| A0A0Y0AXD6 | Actin (Fragment) OS=Panax ginseng OX=4054 GN=ACT PE=2 SV=1 - [A0A0Y0AXD6_PANGI] | 583.64 | 41.14 | 2 | 1 | 10 | 15 | 367 | 40.6 | 5.71 |
| Q1HGF4 | Dehydrin 4 OS=Panax ginseng OX=4054 GN=Dhn4 PE=2 SV=1 - [Q1HGF4_PANGI] | 555.82 | 57.80 | 2 | 13 | 13 | 20 | 218 | 24.5 | 4.98 |
| A0A060IGE0 | Tubulin beta chain (Fragment) OS=Panax ginseng OX=4054 GN=bTUB PE=2 SV=1 - [A0A060IGE0_PANGI] | 552.10 | 44.67 | 1 | 11 | 11 | 16 | 300 | 33.4 | 5.35 |
| A0A060ID32 | Tubulin alpha chain OS=Panax ginseng OX=4054 GN=TUBa PE=2 SV=1 - [A0A060ID32_PANGI] | 495.80 | 22.94 | 1 | 9 | 9 | 13 | 449 | 49.6 | 5.10 |
| A0A096VPN4 | Pleiotropic drug resistance transporter 2 OS=Panax ginseng OX=4054 GN=PDR2 PE=2 SV=1 - [A0A096VPN4_PANGI] | 491.95 | 6.64 | 2 | 1 | 10 | 14 | 1445 | 162.0 | 7.11 |
| A0A060IGT8 | Putative polyubiquitin OS=Panax ginseng OX=4054 GN=UBQ PE=2 SV=1 - [A0A060IGT8_PANGI] | 482.71 | 32.47 | 1 | 6 | 6 | 18 | 154 | 17.2 | 6.06 |
| Q20BN2 | ADP-ribosylation factor-like protein OS=Panax ginseng OX=4054 PE=2 SV=1 - [Q20BN2_PANGI] | 458.52 | 42.54 | 1 | 1 | 6 | 10 | 181 | 20.6 | 7.50 |
| S5RMQ6 | Sesquiterpene synthase OS=Panax ginseng OX=4054 GN=STS PE=2 SV=1 - [S5RMQ6_PANGI] | 443.63 | 18.49 | 1 | 9 | 9 | 13 | 568 | 65.5 | 6.44 |
| A5YU16 | Peptidyl-prolyl cis-trans isomerase OS=Panax ginseng OX=4054 PE=2 SV=1 - [A5YU16_PANGI] | 434.05 | 29.07 | 1 | 5 | 5 | 10 | 172 | 18.0 | 9.09 |
| A0A060ID18 | ADP-ribosylation factor (Fragment) OS=Panax ginseng OX=4054 GN=ARF PE=2 SV=1 - [A0A060ID18_PANGI] | 391.28 | 59.68 | 1 | 2 | 7 | 10 | 124 | 14.4 | 5.81 |
| B5THI3 | Major latex-like protein OS=Panax ginseng OX=4054 GN=mlp151 PE=1 SV=1 - [B5THI3_PANGI] | 388.25 | 64.24 | 1 | 7 | 7 | 15 | 151 | 16.9 | 5.00 |
| P80889 | Ribonuclease 1 OS=Panax ginseng OX=4054 PE=1 SV=1 - [RNS1_PANGI] | 385.47 | 57.14 | 2 | 6 | 6 | 9 | 154 | 16.4 | 4.56 |
| A0A076JQL6 | Pathogenesis-related protein 10 OS=Panax ginseng OX=4054 PE=2 SV=1 - [A0A076JQL6_PANGI] | 341.06 | 48.10 | 1 | 8 | 8 | 11 | 158 | 17.8 | 6.11 |
| E2FGZ1 | Ribulose bisphosphate carboxylase large chain (Fragment) OS=Panax ginseng OX=4054 GN=rbcL PE=3 SV=1 - [E2FGZ1_PANGI] | 336.42 | 27.21 | 9 | 8 | 8 | 12 | 305 | 34.2 | 8.10 |
| O47412 | ATP synthase subunit alpha OS=Panax ginseng OX=4054 GN=atpA PE=3 SV=1 - [O47412_PANGI] | 334.89 | 20.12 | 3 | 9 | 9 | 10 | 507 | 54.9 | 6.24 |
| Q401B8 | 40S ribosomal protein S4 OS=Panax ginseng OX=4054 PE=2 SV=1 - [Q401B8_PANGI] | 328.28 | 37.50 | 1 | 10 | 10 | 11 | 264 | 29.8 | 10.23 |
| A0A0Y0AX85 | Elongation factor 1-beta OS=Panax ginseng OX=4054 GN=EF1-beta PE=2 SV=1 - [A0A0Y0AX85_PANGI] | 301.53 | 36.73 | 1 | 7 | 7 | 10 | 226 | 24.6 | 4.63 |
| A0A060IGR7 | Elongation factor 1-alpha (Fragment) OS=Panax ginseng OX=4054 GN=EF PE=2 SV=1 - [A0A060IGR7_PANGI] | 273.35 | 30.62 | 1 | 8 | 8 | 10 | 258 | 28.3 | 8.46 |
| Q1HGF1 | Dehydrin 7 OS=Panax ginseng OX=4054 GN=Dhn7 PE=2 SV=1 - [Q1HGF1_PANGI] | 265.10 | 24.49 | 2 | 2 | 3 | 7 | 196 | 20.4 | 7.12 |
| P80890 | Ribonuclease 2 OS=Panax ginseng OX=4054 PE=1 SV=1 - [RNS2_PANGI] | 246.15 | 39.87 | 4 | 5 | 5 | 8 | 153 | 16.5 | 4.70 |
| U5NNF1 | Glutathione peroxidase OS=Panax ginseng OX=4054 PE=2 SV=1 - [U5NNF1_PANGI] | 225.33 | 22.57 | 1 | 3 | 4 | 7 | 226 | 25.7 | 6.55 |
| Q20BN0 | BBE domain-containing protein OS=Panax ginseng OX=4054 PE=2 SV=1 - [Q20BN0_PANGI] | 223.35 | 43.79 | 1 | 4 | 4 | 6 | 153 | 17.5 | 8.37 |
| Q68RZ7 | Acetyl-coenzyme A carboxylase carboxyl transferase subunit beta, chloroplastic OS=Panax ginseng OX=4054 GN=accD PE=3 SV=2 - [ACCD_PANGI] | 209.85 | 13.14 | 2 | 7 | 7 | 7 | 487 | 55.1 | 5.06 |
| A0A220QKD0 | Ascorbate peroxidase OS=Panax ginseng OX=4054 PE=2 SV=1 - [A0A220QKD0_PANGI] | 192.36 | 18.80 | 2 | 1 | 4 | 5 | 250 | 27.6 | 5.83 |
| A0A220QKC7 | Ascorbate peroxidase OS=Panax ginseng OX=4054 PE=2 SV=1 - [A0A220QKC7_PANGI] | 189.24 | 28.80 | 2 | 2 | 5 | 7 | 250 | 27.5 | 5.99 |
| U5NNJ1 | Glutathione peroxidase OS=Panax ginseng OX=4054 PE=2 SV=1 - [U5NNJ1_PANGI] | 175.75 | 23.33 | 1 | 4 | 5 | 6 | 240 | 26.3 | 9.06 |
| Q9MAV9 | Cytoplasmic ribosomal protein S13 OS=Panax ginseng OX=4054 PE=2 SV=1 - [Q9MAV9_PANGI] | 175.61 | 28.48 | 1 | 4 | 4 | 5 | 151 | 17.1 | 10.39 |
| O22668 | Superoxide dismutase [Cu-Zn] OS=Panax ginseng OX=4054 GN=SODCC PE=2 SV=1 - [SODC_PANGI] | 172.05 | 21.71 | 1 | 3 | 3 | 7 | 152 | 15.2 | 5.83 |
| A9Z0Q0 | Catalase OS=Panax ginseng OX=4054 GN=Cat1 PE=2 SV=1 - [A9Z0Q0_PANGI] | 161.46 | 12.40 | 1 | 5 | 5 | 6 | 492 | 56.7 | 7.18 |
| I7CT85 | Protopanaxadiol 6-hydroxylase OS=Panax ginseng OX=4054 GN=CYP716A53v2 PE=1 SV=1 - [C7A53_PANGI] | 150.47 | 11.30 | 1 | 5 | 5 | 5 | 469 | 53.3 | 9.01 |
| A0A060ILK6 | Putative V-type proton ATPase subunit B 1 (Fragment) OS=Panax ginseng OX=4054 GN=V-ATP PE=2 SV=1 - [A0A060ILK6_PANGI] | 149.82 | 25.76 | 1 | 4 | 4 | 5 | 132 | 15.0 | 5.08 |
| A0A060IGE8 | 60S ribosomal protein L13a (Fragment) OS=Panax ginseng OX=4054 GN=60s PE=2 SV=1 - [A0A060IGE8_PANGI] | 129.30 | 18.47 | 1 | 4 | 4 | 5 | 157 | 17.8 | 10.65 |
| A9QMA1 | Thioredoxin h-like protein (Fragment) OS=Panax ginseng OX=4054 PE=2 SV=1 - [A9QMA1_PANGI] | 123.55 | 20.31 | 1 | 2 | 2 | 3 | 128 | 14.1 | 5.78 |
| A0A060IMQ8 | Eukaryotic translation initiation factor 5A OS=Panax ginseng OX=4054 GN=eIF-5A PE=2 SV=1 - [A0A060IMQ8_PANGI] | 113.37 | 16.98 | 1 | 3 | 3 | 4 | 159 | 17.2 | 5.78 |
| A0A096XF70 | NADH dehydrogenase subunit 7 OS=Panax ginseng OX=4054 GN=nad7 PE=3 SV=1 - [A0A096XF70_PANGI] | 109.27 | 5.08 | 1 | 2 | 2 | 2 | 394 | 44.2 | 7.39 |
| A9QXE9 | Glutaredoxin OS=Panax ginseng OX=4054 PE=2 SV=1 - [A9QXE9_PANGI] | 109.24 | 32.08 | 1 | 3 | 3 | 3 | 106 | 11.2 | 8.28 |
| A0A3S9H6N0 | Cytochrome P450 CYP82D176 OS=Panax ginseng OX=4054 GN=CYP82D176 PE=2 SV=1 - [A0A3S9H6N0_PANGI] | 99.52 | 4.19 | 1 | 2 | 2 | 2 | 525 | 59.5 | 8.87 |
| Q4JHN6 | Farnesyl pyrophosphate synthase OS=Panax ginseng OX=4054 GN=FPS PE=2 SV=1 - [FPS_PANGI] | 97.62 | 14.33 | 1 | 4 | 4 | 4 | 342 | 39.6 | 5.92 |
| A0A096XF79 | NADH dehydrogenase subunit 9 OS=Panax ginseng OX=4054 GN=nad9 PE=3 SV=1 - [A0A096XF79_PANGI] | 97.21 | 12.11 | 1 | 2 | 2 | 2 | 190 | 22.5 | 7.50 |
| Q1HGF5 | Dehydrin 3 OS=Panax ginseng OX=4054 GN=Dhn3 PE=2 SV=1 - [Q1HGF5_PANGI] | 95.29 | 65.63 | 1 | 4 | 6 | 7 | 96 | 10.2 | 7.21 |
| A0A096XF91 | ATPase subunit 8 OS=Panax ginseng OX=4054 GN=atp8 PE=4 SV=1 - [A0A096XF91_PANGI] | 94.97 | 13.21 | 1 | 3 | 3 | 3 | 159 | 18.2 | 9.03 |
| A0A060ID14 | Cyclophilin (Fragment) OS=Panax ginseng OX=4054 GN=CYP PE=2 SV=1 - [A0A060ID14_PANGI] | 93.19 | 9.52 | 1 | 1 | 1 | 2 | 84 | 9.0 | 9.92 |
| A0A060ILM1 | Ubiquinol-cytochrome C reductase iron-sulfur subunit OS=Panax ginseng OX=4054 GN=QCR PE=2 SV=1 - [A0A060ILM1_PANGI] | 87.63 | 15.66 | 1 | 3 | 3 | 4 | 281 | 30.7 | 8.65 |
| Q1HGF6 | Dehydrin 2 OS=Panax ginseng OX=4054 GN=Dhn2 PE=2 SV=1 - [Q1HGF6_PANGI] | 86.49 | 63.37 | 1 | 2 | 4 | 10 | 101 | 10.8 | 7.50 |
| A0A096XF73 | ATPase subunit 4 OS=Panax ginseng OX=4054 GN=atp4 PE=4 SV=1 - [A0A096XF73_PANGI] | 82.09 | 10.42 | 1 | 2 | 2 | 2 | 192 | 21.3 | 9.69 |
| H2DH23 | Cytochrome P450 CYP82H23 (Fragment) OS=Panax ginseng OX=4054 PE=2 SV=1 - [C7H23_PANGI] | 80.32 | 8.16 | 1 | 2 | 2 | 2 | 245 | 28.1 | 8.51 |
| A0A096XF77 | Cytochrome b OS=Panax ginseng OX=4054 GN=cob2 PE=3 SV=1 - [A0A096XF77_PANGI] | 79.68 | 5.60 | 1 | 2 | 2 | 2 | 393 | 43.9 | 7.72 |
| A0A060IMP7 | Putative GTP-binding protein sar1 (Fragment) OS=Panax ginseng OX=4054 GN=SAR PE=2 SV=1 - [A0A060IMP7_PANGI] | 74.28 | 46.15 | 1 | 2 | 2 | 3 | 65 | 7.4 | 7.52 |
| Q8RVT5 | Acyl-CoA-binding protein OS=Panax ginseng OX=4054 GN=ACBP PE=2 SV=1 - [Q8RVT5_PANGI] | 71.98 | 27.59 | 1 | 2 | 2 | 2 | 87 | 9.9 | 5.50 |
| D7RWP9 | Peroxiredoxin OS=Panax ginseng OX=4054 PE=2 SV=1 - [D7RWP9_PANGI] | 71.30 | 10.49 | 1 | 2 | 2 | 2 | 162 | 17.4 | 5.59 |
| Q68RY2 | ATP-dependent Clp protease proteolytic subunit OS=Panax ginseng OX=4054 GN=clpP PE=3 SV=1 - [CLPP_PANGI] | 71.21 | 7.65 | 1 | 1 | 1 | 1 | 196 | 22.1 | 4.83 |
| C1KGC1 | Calmodulin OS=Panax ginseng OX=4054 GN=Cam PE=2 SV=1 - [C1KGC1_PANGI] | 68.68 | 10.74 | 1 | 1 | 1 | 1 | 149 | 16.8 | 4.27 |
| Q307T0 | Tonoplast intrinsic protein OS=Panax ginseng OX=4054 GN=TIP1 PE=2 SV=1 - [Q307T0_PANGI] | 68.67 | 8.00 | 1 | 2 | 2 | 3 | 250 | 25.6 | 5.95 |
| Q1HGF2 | Dehydrin 6 (Fragment) OS=Panax ginseng OX=4054 GN=Dhn6 PE=2 SV=1 - [Q1HGF2_PANGI] | 67.69 | 22.02 | 1 | 2 | 3 | 4 | 168 | 17.0 | 6.54 |
| A0A068ENE1 | Signal peptidase complex subunit 3B (Fragment) OS=Panax ginseng OX=4054 PE=4 SV=1 - [A0A068ENE1_PANGI] | 60.75 | 15.79 | 3 | 1 | 1 | 1 | 57 | 6.6 | 9.47 |
| A0A060L489 | NADPH--cytochrome P450 reductase OS=Panax ginseng OX=4054 GN=CPR2 PE=2 SV=1 - [A0A060L489_PANGI] | 59.15 | 3.69 | 3 | 2 | 2 | 2 | 678 | 75.4 | 5.43 |
| A0A060IMR4 | Translationally controlled tumor protein OS=Panax ginseng OX=4054 GN=TCTP PE=2 SV=1 - [A0A060IMR4_PANGI] | 58.49 | 5.36 | 1 | 1 | 1 | 1 | 168 | 18.9 | 4.70 |
| B2MVQ2 | Glutathione S-transferase OS=Panax ginseng OX=4054 GN=GST PE=2 SV=1 - [B2MVQ2_PANGI] | 55.17 | 10.00 | 1 | 2 | 2 | 2 | 250 | 28.3 | 9.03 |
| A0A0A6ZFY4 | UDP-glucosyltransferase 29 OS=Panax ginseng OX=4054 GN=UGT29 PE=1 SV=1 - [UGT29_PANGI] | 46.72 | 2.71 | 2 | 1 | 1 | 1 | 442 | 49.1 | 5.92 |
| A0A3S9H6J5 | Cytochrome P450 CYP82D175 OS=Panax ginseng OX=4054 GN=CYP82D175 PE=2 SV=1 - [A0A3S9H6J5_PANGI] | 42.52 | 1.34 | 1 | 1 | 1 | 1 | 522 | 59.2 | 8.03 |
| B6DMH0 | Cinnamyl alcohol dehydrogenase (Fragment) OS=Panax ginseng OX=4054 PE=2 SV=1 - [B6DMH0_PANGI] | 42.43 | 5.32 | 1 | 1 | 1 | 1 | 188 | 20.1 | 8.34 |
| A0A290GN01 | Superoxide dismutase (Fragment) OS=Panax ginseng OX=4054 GN=MSD1 PE=2 SV=1 - [A0A290GN01_PANGI] | 41.25 | 9.27 | 1 | 1 | 1 | 1 | 151 | 16.1 | 6.95 |
| B8YDG5 | Short-chain alcohol dehydrogenase OS=Panax ginseng OX=4054 PE=2 SV=1 - [B8YDG5_PANGI] | 40.12 | 8.27 | 1 | 1 | 1 | 1 | 266 | 29.0 | 7.33 |
| A0A3Q9BGP9 | Cytochrome P450 CYP80F5 OS=Panax ginseng OX=4054 GN=CYP80F5 PE=2 SV=1 - [A0A3Q9BGP9_PANGI] | 39.92 | 9.76 | 1 | 3 | 3 | 3 | 502 | 56.4 | 7.93 |
| Q5I681 | Calcium-dependent/calmodulin-independent protein kinase (Fragment) OS=Panax ginseng OX=4054 PE=2 SV=1 - [Q5I681_PANGI] | 39.56 | 6.96 | 1 | 1 | 1 | 1 | 273 | 30.6 | 4.75 |
| C1K2M3 | Class 1 chitinase OS=Panax ginseng OX=4054 GN=Chi-1 PE=2 SV=1 - [C1K2M3_PANGI] | 37.10 | 4.64 | 1 | 1 | 1 | 1 | 323 | 34.9 | 8.21 |
| Q3LFQ4 | Ribulose bisphosphate carboxylase small chain OS=Panax ginseng OX=4054 PE=2 SV=1 - [Q3LFQ4_PANGI] | 35.26 | 4.37 | 1 | 1 | 1 | 1 | 183 | 20.5 | 8.32 |
| A9QMA3 | 60S ribosomal protein L17-like protein (Fragment) OS=Panax ginseng OX=4054 PE=2 SV=1 - [A9QMA3_PANGI] | 33.79 | 18.84 | 1 | 3 | 3 | 3 | 138 | 14.8 | 10.48 |
| Q68S04 | 30S ribosomal protein S4, chloroplastic OS=Panax ginseng OX=4054 GN=rps4 PE=3 SV=1 - [RR4_PANGI] | 30.64 | 5.47 | 1 | 1 | 1 | 1 | 201 | 23.3 | 10.67 |
| A0A3Q9BGT6 | Cytochrome P450 CYP81B102 OS=Panax ginseng OX=4054 GN=CYP81B102 PE=2 SV=1 - [A0A3Q9BGT6_PANGI] | 29.15 | 2.17 | 1 | 1 | 1 | 1 | 508 | 58.3 | 8.75 |
| A0A0D5ZCT2 | Glycosyltransferase OS=Panax ginseng OX=4054 PE=2 SV=1 - [A0A0D5ZCT2_PANGI] | 28.60 | 1.63 | 1 | 1 | 1 | 1 | 491 | 54.7 | 5.45 |
| A0A0K0PVL3 | UDP-glucosyltransferase 103 OS=Panax ginseng OX=4054 GN=UGT103 PE=1 SV=1 - [UGT13_PANGI] | 28.50 | 1.48 | 6 | 1 | 1 | 1 | 472 | 53.2 | 5.48 |
| A0A0Y0C5Z0 | Eukaryotic translation initiation factor 3 subunit B (Fragment) OS=Panax ginseng OX=4054 GN=IF3B PE=2 SV=1 - [A0A0Y0C5Z0_PANGI] | 26.42 | 2.53 | 1 | 1 | 1 | 1 | 356 | 40.3 | 5.05 |
| A0A0D5ZDF8 | Glycosyltransferase OS=Panax ginseng OX=4054 PE=2 SV=1 - [A0A0D5ZDF8_PANGI] | 24.09 | 2.10 | 1 | 1 | 1 | 1 | 477 | 53.9 | 6.06 |
| Q6JSK3 | Betaine aldehyde dehydrogenase OS=Panax ginseng OX=4054 GN=BADH1 PE=2 SV=1 - [Q6JSK3_PANGI] | 23.52 | 2.39 | 1 | 1 | 1 | 1 | 503 | 54.6 | 5.44 |
| Q9MAW6 | 60S ribosomal protein L27a OS=Panax ginseng OX=4054 PE=2 SV=1 - [Q9MAW6_PANGI] | 22.88 | 12.33 | 1 | 1 | 1 | 1 | 146 | 16.3 | 10.48 |
| A0A166JH20 | Peptidyl-prolyl cis-trans isomerase OS=Panax ginseng OX=4054 PE=2 SV=1 - [A0A166JH20_PANGI] | 15.52 | 15.52 | 1 | 2 | 2 | 2 | 174 | 18.7 | 7.81 |
| A0A0U3JLF4 | 26S proteasome non-ATPase regulatory subunit 14-like protein (Fragment) OS=Panax ginseng OX=4054 PE=4 SV=1 - [A0A0U3JLF4_PANGI] | 14.21 | 29.03 | 3 | 1 | 1 | 1 | 31 | 3.7 | 9.70 |
| A0A0Y0AU34 | Elongation factor 1-gamma (Fragment) OS=Panax ginseng OX=4054 GN=EF1-gamma PE=2 SV=1 - [A0A0Y0AU34_PANGI] | 0.00 | 12.55 | 1 | 2 | 2 | 2 | 239 | 26.5 | 8.50 |

**Additional file 1: Table S5. Readcount_TPM**

| **sRNA.readcount** | **renshen.readcount** | **renshen.tpm** |
| --- | --- | --- |
| aau-miR160 | 12 | 42.72956 |
| aof-miR5139b | 1 | 42.72956 |
| aqc-miR159 | 34 | 42.72956 |
| ath-miR156a-5p | 11 | 42.72956 |
| ath-miR157a-5p | 15 | 42.72956 |
| ath-miR159a | 457 | 42.72956 |
| ath-miR159b-3p | 457 | 42.72956 |
| ath-miR159c | 457 | 42.72956 |
| ath-miR160a-5p | 12 | 42.72956 |
| ath-miR164a | 12 | 42.72956 |
| ath-miR164c-5p | 8 | 85.45913 |
| ath-miR166a-3p | 197 | 85.45913 |
| ath-miR172c | 1 | 85.45913 |
| ath-miR319a | 67 | 85.45913 |
| ath-miR319c | 67 | 128.1887 |
| ath-miR394a | 11 | 128.1887 |
| ath-miR396a-3p | 247 | 170.9183 |
| ath-miR396a-5p | 1214 | 170.9183 |
| ath-miR396b-5p | 1364 | 170.9183 |
| ath-miR403-3p | 43 | 170.9183 |
| atr-miR396d | 10 | 213.6478 |
| bdi-miR159a-3p | 59 | 213.6478 |
| bdi-miR845 | 3 | 213.6478 |
| cca-miR156b | 5 | 213.6478 |
| cca-miR396a-3p | 5 | 256.3774 |
| cca-miR396c | 4 | 299.107 |
| cme-miR166i | 1 | 299.107 |
| cpa-miR166e | 194 | 299.107 |
| cpa-miR8155 | 1 | 341.8365 |
| csi-miR156d-5p | 14 | 341.8365 |
| csi-miR160a-5p | 12 | 384.5661 |
| csi-miR160c-5p | 2 | 427.2956 |
| csi-miR396a-3p | 23 | 427.2956 |
| csi-miR396e-5p | 1687 | 470.0252 |
| csi-miR403b-5p | 4 | 470.0252 |
| fve-miR156h | 5 | 470.0252 |
| fve-miR159b | 61 | 512.7548 |
| fve-miR159c | 61 | 512.7548 |
| gma-miR160b | 12 | 512.7548 |
| gma-miR164b | 12 | 512.7548 |
| gma-miR166m | 1 | 512.7548 |
| gma-miR166u | 198 | 512.7548 |
| gma-miR319c | 67 | 512.7548 |
| gma-miR319q | 67 | 512.7548 |
| gma-miR396a-3p | 247 | 555.4843 |
| gma-miR396h | 1214 | 598.2139 |
| gma-miR403a | 4 | 640.9435 |
| gma-miR4995 | 6 | 640.9435 |
| gma-miR5368 | 12 | 726.4026 |
| gma-miR6300 | 11 | 982.78 |
| han-miR3630-3p | 2 | 1153.698 |
| hbr-miR156 | 15 | 1452.805 |
| hci-miR164a | 1 | 1837.371 |
| lja-miR166-3p | 201 | 2521.044 |
| lus-miR159b | 457 | 2521.044 |
| mdm-miR159a | 59 | 2521.044 |
| mdm-miR396a | 1172 | 2521.044 |
| mtr-miR166b | 7 | 2521.044 |
| mtr-miR319a-3p | 67 | 2521.044 |
| osa-miR159a.1 | 457 | 2606.503 |
| osa-miR159c | 61 | 2606.503 |
| osa-miR159d | 61 | 2606.503 |
| osa-miR159e | 61 | 2606.503 |
| osa-miR159f | 59 | 2606.503 |
| osa-miR160e-5p | 12 | 2862.881 |
| osa-miR164d | 8 | 2862.881 |
| osa-miR164e | 1 | 2862.881 |
| osa-miR166g-3p | 10 | 2862.881 |
| osa-miR166i-3p | 9 | 2862.881 |
| osa-miR166m | 27 | 2905.61 |
| osa-miR396a-3p | 104 | 2905.61 |
| pde-miR396 | 17 | 4443.875 |
| peu-miR2916 | 2 | 4443.875 |
| pgi-miR482a | 3 | 8289.536 |
| ppe-miR1511-3p | 1 | 8417.724 |
| ppe-miR396a | 1170 | 8417.724 |
| ppt-miR166j | 2 | 8460.454 |
| ppt-miR319a | 68 | 8588.642 |
| pta-miR159a | 59 | 10554.2 |
| pta-miR159c | 59 | 10554.2 |
| pta-miR319 | 68 | 19527.41 |
| ptc-miR166n | 7 | 19527.41 |
| ptc-miR396f | 2974 | 19527.41 |
| ptc-miR396g-5p | 1687 | 19527.41 |
| rgl-miR5139 | 1 | 19527.41 |
| sly-miR403-5p | 4 | 49993.59 |
| smo-miR396 | 1577 | 50079.05 |
| sof-miR159c | 59 | 51104.56 |
| stu-miR156f-5p | 5 | 51873.69 |
| vvi-miR166a | 7 | 51873.69 |
| vvi-miR3630-3p | 13 | 51873.69 |
| vvi-miR396a | 1196 | 58283.13 |
| vvi-miR396b | 1368 | 58454.04 |
| zma-miR164h-5p | 1 | 67384.52 |
| zma-miR166h-3p | 197 | 72084.78 |
| zma-miR396g-3p | 1214 | 72084.78 |
| zma-miR396g-5p | 104 | 127077.7 |
